# Supplementary material for: Marked difference in liver fat measured by histology vs. magnetic resonance-proton density fat fraction: A meta-analysis
Source: JHEP Rep. 2023 Oct 11;6(1):100928. doi: 10.1016/j.jhepr.2023.100928 (PMC10711480; doi:10.1016/j.jhepr.2023.100928)
Supplement: Multimedia component 1 [file mmc1.pdf]

Marked difference in liver fat measured by histology *vs.* magnetic resonance-proton density fat fraction: A meta-analysis

Sami Qadri, Emilia Vartiainen, Mari Lahelma, Kimmo Porthan, An Tang, Ilkay S. Idilman, Jurgen H. Runge, Anne Juuti, Anne K. Penttilä, Juhani Dabek, Tiina E. Lehtimäki, Wenla Seppänen, Johanna Arola, Perttu Arkkila, Jaap Stoker, Musturay Karcaaltincaba, Michael Pavlides, Rohit Loomba, Claude B. Sirlin, Taru Tukiainen, Hannele Yki-Järvinen

Table of contents

Supplementary Methods 2
The Helsinki MRS-PDFF cohort 2
The liver RNA-seq cohort 3
Supplementary Tables 5
Table S1 5
Table S2 7
Table S3 8
Table S4 9
Table S5 10
Table S6 11
Table S7 12
Supplementary Figures 13
Fig. S1 13
Fig. S2 14
Fig. S3 15
Fig. S4 16
Fig. S5 17
Fig. S6 18
Fig. S7 19
Supplementary References 20

Supplementary Methods

The Helsinki MRS-PDFF cohort

Patients and study design

We recruited 71 consecutive patients undergoing metabolic surgery at the Helsinki University Hospital (Helsinki, Finland), who also underwent a liver biopsy in conjunction with liver fat measurement by MRS-PDFF. All patients fulfilled the following inclusion criteria: (i) age 18 to 75 years; (ii) no evidence of primary liver diseases other than NAFLD based on history, physical examination, and standard laboratory tests (including assays for hepatitis B virus surface antigen, hepatitis A and C virus antibodies, anti-smooth muscle antibodies, anti-mitochondrial antibodies, and anti-nuclear antibodies); (iii) alcohol consumption less than 20/30 g per day for females/males; (iv) no use of drugs or toxins associated with liver steatosis; and (v) not pregnant or lactating. Approximately a week before undergoing a liver biopsy, the patients arrived at the Clinical Research Unit after an overnight fast. A history and physical examination were performed, including measurement of body weight and height, as well as blood sampling for biochemical measurements. Spectroscopic assessment of liver fat was conducted within approximately a week of the liver biopsy. After explaining the potential risks associated with the study, all patients provided a written informed consent for their participation. The Regional Ethics Committee of the Hospital District of Helsinki and Uusimaa (Helsinki, Finland) approved the study protocol, and the study was conducted in accordance with the Declaration of Helsinki.

Liver histology

Liver biopsies were processed and stained using routine protocols of the Department of Pathology. Histopathology was assessed by an experienced hepatopathologist (J.A.), blinded to the clinical data and MRS-PDFF results.[1] Liver fat was determined as the fraction of lobular hepatocytes containing macrovesicular lipid droplets (*i.e.*, large inclusions of lipid that displace the nucleus to the cell's periphery). NASH was diagnosed when steatosis, inflammation, and ballooning were concomitantly present.[2]

Magnetic resonance spectroscopy

After a minimum of 4 hours of fasting, liver fat content was measured by ^{1}H-MRS using the 1.5T GE Signa HDxt MRI scanner (GE Medical Systems, Waukesha, WI). The point resolved spectroscopy sequence was used with an echo time (TE) of 30 ms, repetition time (TR) of 3000 ms, and with 1024 data points over 1000 kHz spectral width and 16 acquisitions. Prior

to MRS measurements, T1-weighted localization images were acquired using a standard ^1H body coil. A 27 cm^3 voxel was then carefully positioned in the right lobe of the liver avoiding large vessels, bile ducts, and the gallbladder. Subjects were allowed to breathe freely during the data collection. All spectra were analyzed by a single investigator with the jMRUI v5.2 software using the AMARES algorithm.[3] Intensities of the spectral peaks resonating from protons of water (water peak, 3–6 ppm) and protons of methylene or methyl groups in fatty acid chains (fat peak, 0.5–3 ppm) were determined using line-shape fitting with prior knowledge. Signal intensities were corrected using the equation $I_m = I_0 exp(-TE/T2)$, and T2 values of 46 ms and 58 ms were used for water and fat. Signal intensities were corrected for T2 relaxation. T1 corrections were not needed due to the long TR used. To account for the spectral complexity of liver fat, we employed an internally developed fat model which accounts for—in addition to signal originating from the fat peak—signal originating from triglyceride-associated protons underlying the water peak: the methine group peak in fatty acids (carbon–carbon double bonds with single protons, at 5.28–5.46 ppm), and the glycerol group peaks of the single proton (at 5.27 ppm) and of the double protons (at 4.22 ppm). The mathematical fat model was generated based on biochemically determined lipid composition of 125 human liver tissue samples using ultra-high performance liquid chromatography coupled with mass spectrometry. Finally, liver fat content (PDFF) was calculated as the ratio of signal from mobile protons in triglycerides to the sum of signal from mobile protons in triglycerides and free water.

The liver RNA-seq cohort

Using an identical protocol and inclusion/exclusion criteria as described above for the Helsinki MRS-PDFF cohort, we recruited a separate cohort of 138 individuals undergoing metabolic surgery at the Helsinki University Hospital. The patients similarly underwent a clinical study approximately a week of the liver biopsy. An intraoperative wedge biopsy of the liver was obtained during laparoscopy, and part of the biopsy was sent to an experienced hepatopathologist (J.A.) for histological evaluation as described above. Another part was immediately snap-frozen in liquid nitrogen for subsequent analysis of the hepatic transcriptome. The Regional Ethics Committee of the Hospital District of Helsinki and Uusimaa (Helsinki, Finland) approved the study protocol, and the study was conducted in accordance with the Declaration of Helsinki.

Liver transcriptomic analysis

Tissue RNA was extracted from the 138 liver biopsies using the AllPrep DNA/RNA/miRNA Universal Kit (QIAGEN, Hilden, Germany). Libraries were constructed using Illumina stranded mRNA library preparation (Illumina, San Diego, CA), and the samples underwent bulk RNA

sequencing using paired-end 150 bp reads on an Illumina platform. Before filtering, the number of reads was 100 M per sample. Our RNA-seq analysis pipeline closely followed the Genotype-Tissue Expression (GTEx) V8 RNA-seq analysis pipeline, with minor extensions and modifications.[4] After quality control and adapter trimming, STAR version 2.6.0a was used to align output reads to the human reference genome GRCh38/hg38.[5] Genes were annotated with STAR using GENCODE 26 transcript model annotation. Gene-level expression was calculated based on a collapsed gene model, where all isoforms were collapsed to a single transcript per gene. Read counts and transcripts per million (TPM) values were then produced using RNA-SeQC version 2.0.3.[6]

Cell-type decomposition analysis

Cell-type decomposition analysis was performed using CIBERSORTx to resolve proportions of distinct cell populations in bulk liver tissue expression profiles by using signature genes derived from a previously published human liver single-cell RNA-seq dataset.[7, 8] Default parameters were used in creating the signature matrix from single-cell RNA-seq data and computing of cell fractions using the Cell Fractions module. Batch correction was enabled with S-mode, quantile normalization was disabled, and the number of permutations was set to 100.

Supplementary Tables

Table S1. Electronic search strategy for the systematic review, which was conducted in all databases on August 16, 2022.

Search number	Query	Results
	**PubMed**	
1	"Fatty Liver"[Mesh] OR Fatty Liver*[tiab] OR Liver Fat*[tiab] OR Fat Of Liver*[tiab] OR Fat Of The Liver*[tiab] OR Fats Of Liver*[tiab] OR Fats Of The Liver*[tiab] OR Liver Steatos*[tiab] OR Steatosis Liver*[tiab] OR Steatoses Liver*[tiab] OR Steatosis Of Liver*[tiab] OR Steatosis Of The Liver*[tiab] OR Steatoses Of Liver*[tiab] OR Steatoses Of The Liver*[tiab] OR Steatohepatiti*[tiab] OR Hepatosteatos*[tiab] OR Hepatic Steatos*[tiab] OR Hepatocellular Steatos*[tiab] OR Hepatocellular Fat*[tiab] OR Hepatic Fat*[tiab] OR NAFLD[tiab] OR MAFLD[tiab] OR NASH[tiab]	73,053
2	Pathologists[Mesh] OR Pathologist*[tiab] OR Biopsy[Mesh] OR Biops*[tiab] OR Histology[Mesh] OR Histolog*[tiab]	1,441,674
3	"Magnetic Resonance Imaging"[Mesh] OR "Magnetic Resonance"[tiab] OR MRI[tiab] OR "Magnetic Resonance Spectroscopy"[Mesh] OR Spectroscop*[tiab] OR MRS[tiab] OR Magnetic Imag*[tiab] OR MR Imag*[tiab] OR PDFF[tiab] OR "Proton Density Fat Fraction"[tiab]	1,307,687
4	#1 AND #2 AND #3	1 137
	**Scopus**	
1	(TITLE-ABS-KEY("Fatty Liver*" OR "Liver* Fat*" OR "Fat* Of Liver*" OR "Fat* Of The Liver*" OR "Liver* Steatos*" OR "Steatos* Liver*" OR "Steatos* Of Liver*" OR "Steatos* Of The Liver*" OR "Steatohepatiti*" OR "Hepatosteatos*" OR "Hepatic Steatos*" OR "Hepatocellular Steatos*" OR "Hepatocellular Fat*" OR "Hepatic Fat*" OR "NAFLD" OR "MAFLD" OR "NASH")) AND (TITLE-ABS-KEY("Pathologist*" OR "Biops*" OR "Histolog*")) AND (TITLE-ABS-KEY("Magnetic Resonance" OR "MRI" OR "Spectroscop*" OR "MRS" OR "Magnetic Imag*" OR "MR Imag*" OR "PDFF" OR "Proton Density Fat Fraction"))	2 616
	**Web of Science**	
1	TS=("Fatty Liver*" OR "Liver* Fat*" OR "Fat* Of Liver*" OR "Fat* Of The Liver*" OR "Liver* Steatos*" OR "Steatos* Liver*" OR "Steatos* Of Liver*" OR "Steatos* Of The Liver*" OR "Steatohepatiti*" OR "Hepatosteatos*" OR "Hepatic Steatos*" OR "Hepatocellular Steatos*" OR "Hepatocellular Fat*" OR "Hepatic Fat*" OR "NAFLD" OR "MAFLD" OR "NASH")	117,285
2	TS=("Pathologist*" OR "Biops*" OR "Histolog*")	909,324

3	TS=("Magnetic Resonance" OR "MRI" OR "Spectroscop*" OR "MRS" OR "Magnetic Imag*" OR "MR Imag*" OR "PDFF" OR "Proton Density Fat Fraction")	2,610,276
4	#1 AND #2 AND #3	1 350
	**Cochrane Library**	
1	MeSH descriptor: [Fatty Liver] explode all trees	1 654
2	("Fatty Liver*" OR "Liver* Fat*" OR "Fat* Of Liver*" OR "Fat* Of The Liver*" OR "Liver* Steatos*" OR "Steatos* Liver*" OR "Steatos* Of Liver*" OR "Steatos* Of The Liver*" OR "Steatohepatiti*" OR "Hepatosteatos*" OR "Hepatic Steatos*" OR "Hepatocellular Steatos*" OR "Hepatocellular Fat*" OR "Hepatic Fat*" OR "NAFLD" OR "MAFLD" OR "NASH")	6 214
3	#1 OR #2	6 216
4	MeSH descriptor: [Pathologists] explode all trees	7
5	MeSH descriptor: [Biopsy] explode all trees	6 025
6	MeSH descriptor: [Histology] explode all trees	1 381
7	("Pathologist*" OR "Biops*" OR "Histolog*")	1 839
8	#4 OR #5 OR #6 OR #7	8 771
9	MeSH descriptor: [Magnetic Resonance Imaging] explode all trees	8 944
10	MeSH descriptor: [Magnetic Resonance Spectroscopy] explode all trees	743
11	("Magnetic Resonance" OR "MRI" OR "Spectroscop*" OR "MRS" OR "Magnetic Imag*" OR "MR Imag*" OR "PDFF" OR "Proton Density Fat Fraction")	46,097
12	#9 OR #10 OR #11	46,197
13	#3 AND #8 AND #12 in Trials	24

Table S2. Clinical characteristics of the Helsinki MRS-PDFF cohort.

	**All patients (n = 71)**
Age, years	52 ± 11
Males, n (%)	21 (29.6)
BMI, kg/m^2	37.6 [32.9–41.2]
fP-Glucose, mmol/L	6.4 ± 1.6
B-HbA$_{1c}$, %	6.0 ± 1.0
fP-Total cholesterol, mmol/L	4.3 ± 1.1
fP-HDL cholesterol, mmol/L	1.17 ± 0.38
fP-LDL cholesterol, mmol/L	2.8 ± 1.0
fP-Triglycerides, mmol/L	1.54 ± 1.29
P-ALT, U/L	48 ± 41
P-AST, U/L	35 ± 20
P-AST/ALT	0.9 ± 0.4
P-GGT, U/L	50 ± 58
P-ALP, U/L	73 ± 24
P-Albumin, g/L	39 ± 3
B-Platelets, E10^9/L	256 ± 57
Type 2 diabetes, n (%)	30 (42.3)
Liver fat by MRS, %	7.2 [2.8–15.7]
Liver fat by histology, %	20 [0–40]
NASH, n (%)	19 (26.7)
Fibrosis stage (F0/F1/F2/F3/F4), n	37/20/8/5/1

Data are shown as *means ± standard deviations*, *medians [25th–75th percentiles]*, *counts (percentages)*, or *counts*. Abbreviations: ALP, alkaline phosphatase; ALT, alanine aminotransferase; AST, aspartate aminotransferase; B, blood; BMI, body mass index; f, fasting; GGT, gamma-glutamyltransferase; HbA$_{1C}$, glycated hemoglobin A$_{1C}$; HDL, high-density lipoprotein; LDL, low-density lipoprotein; MRS, magnetic resonance spectroscopy; NASH, nonalcoholic steatohepatitis; P, plasma.

Table S3. Clinical characteristics of the liver RNA-seq cohort.

	**All patients (n = 138)**
Age, years	50 ± 9
Males, n (%)	46 (33.3)
BMI, kg/m^2	42.5 [37.9–46.9]
fP-Glucose, mmol/L	6.1 ± 1.3
B-HbA$_{1c}$, %	6.1 ± 1.0
fP-Total cholesterol, mmol/L	4.1 ± 1.1
fP-HDL cholesterol, mmol/L	1.15 ± 0.28
fP-LDL cholesterol, mmol/L	2.4 ± 0.9
fP-Triglycerides, mmol/L	1.51 ± 1.36
P-ALT, U/L	36 ± 23
P-AST, U/L	32 ± 14
P-AST/ALT	1.0 ± 0.4
P-GGT, U/L	39 ± 36
P-ALP, U/L	65 ± 22
P-Albumin, g/L	38 ± 3
B-Platelets, E10^9/L	251 ± 61
Type 2 diabetes, n (%)	87 (63.0)
Liver fat by histology, %	5 [0–20]
NASH, n (%)	18 (13)
Fibrosis stage (F0/F1/F2/F3/F4), n	76/49/6/6/1

Data are shown as *means ± standard deviations*, *medians [25th–75th percentiles]*, *counts (percentages)*, or *counts*. Abbreviations: ALP, alkaline phosphatase; ALT, alanine aminotransferase; AST, aspartate aminotransferase; B, blood; BMI, body mass index; f, fasting; GGT, gamma-glutamyltransferase; HbA$_{1C}$, glycated hemoglobin A$_{1C}$; HDL, high-density lipoprotein; LDL, low-density lipoprotein; NASH, nonalcoholic steatohepatitis; P, plasma.

Table S4. Methodological details of magnetic resonance protocols used in the selected studies.

Author, year, ref.	Modality	Scanner manufacturer	Field strength	Dimension	Repetition time, ms	Echo time, ms	Number of echoes	Pulse sequence	Reconstruction method	VOIs/ROIs
Qadri, 2022*	MRS-PDFF	GE	1.5 T	N/A	3000	30	1	PRESS	N/A	1 x 27 cm^3
Runge, 2018 [9]	MRS-PDFF	Philips	3.0 T	N/A	3500	10, 15, 20, 25, 30	5	STEAM	N/A	1 x 8 cm^3
Pavlides, 2017 [10]	MRS-PDFF	Siemens	3.0 T	N/A	2000	10	1	N/R	N/A	1 x 8 cm^3
Traussnigg, 2017 [11]	MRS-PDFF	Siemens	3.0 T	N/A	3000	30	1	PRESS	N/A	1 x 27 cm^3
Rastogi, 2016 [12]	MRS-PDFF	Philips	3.0 T	N/A	2000	15, 20, 25, 30, 35	5	STEAM	N/A	1 x 8 cm^3
Tang, 2015 [13]	MRI-PDFF	GE	3.0 T	2D	2000	1.15–6.9	6	N/A	Magnitude	9 x ~3.14 cm^2
Hwang, 2014 [14]	MRS-PDFF	Siemens	3.0 T	N/A	3000	12, 24, 36, 48, 72	5	STEAM	N/A	1 x 27 cm^3
Parente, 2014 [15]	MRS-PDFF	Philips	3.0 T	N/A	2000 / 4000	40, 50, 60, 70, 80, 90, 100, 110	8	PRESS	N/A	1 x 27 cm^3
Idilman, 2013 [16]	MRI-PDFF	GE	1.5 T	3D	3000	1.6–9.8	6	N/A	Complex	1 x 4 cm^2 + 8 x 2 cm^2

Abbreviations: N/A, not applicable; N/R, not reported; PRESS, point-resolved spectroscopy; STEAM, stimulated echo acquisition mode.
*Previously unpublished data from the Helsinki MRS-PDFF cohort.

Table S5. QUADAS-2 quality and risk-of-bias assessment.

Study	Risk of bias				Applicability concerns		
	Patient selection	Index test	Reference standard	Flow and timing	Patient selection	Index test	Reference standard
Qadri 2022	☺	☺	☹	☺	☺	☺	☺
Runge 2018	☺	☺	☹	☺	☺	☺	☺
Pavlides 2017	?	☺	☺	☺	☺	☺	☺
Traussnigg 2017	☺	☺	?	☺	☺	☺	☺
Rastogi 2016	?	☺	☹	☺	☺	☺	☺
Tang 2015	☺	☺	☹	☺	☺	☺	☺
Parente 2014	☺	☺	☹	☺	☺	☺	☺
Hwang 2014	☺	☺	☹	☺	☺	☺	☺
Idilman 2013	☺	☺	☹	☺	☺	☺	☺

☺ Low Risk ☹ High Risk ? Unclear Risk

Table S6. Classification of steatosis grades by histology as compared with PDFF, based on thresholds that are commonly used for histological steatosis grade assessment.

	PDFF				
**Histology**	**S0**	**S1**	**S2**	**S3**	**Total**
**S0**	119	19	0	0	138
**S1**	66	182	0	0	248
**S2**	2	112	2	0	116
**S3**	0	84	11	0	95
**Total**	187	397	13	0	597

Thresholds used to classify steatosis grades: S0: <5%; S1: 5–33%; S2: 34–66%; S3: >66%.

Table S7. Thresholds and raw diagnostic performance parameters for PDFF to predict dichotomized histological steatosis grades at varying sensitivities and specificities in the pooled cohort.

Steatosis grade classification	Threshold	Se, % (95% CI)	Sp, % (95% CI)	PPV, % (95% CI)	NPV, % (95% CI)
**Specificity 90% (rule-in thresholds)**					
S0 vs. S1–S3	≥5.75	79.5 (75.8–83.0)	90.6 (85.5–94.9)	96.6 (94.8–98.2)	57.1 (52.9–61.8)
S0–S1 vs. S2–S3	≥15.50	78.7 (73.0–84.4)	90.2 (87.1–93.0)	81.4 (76.8–86.1)	88.5 (85.9–91.3)
S0–S2 vs. S3	≥21.35	69.5 (60.0–77.9)	90.0 (87.5–92.6)	56.9 (49.6–64.6)	94.0 (92.2–95.7)
**Specificity 95% (rule-in thresholds)**					
S0 vs. S1–S3	≥6.49	76.9 (72.8–80.8)	95.6 (92.0–98.5)	98.3 (97.0–99.4)	55.5 (51.4–60.1)
S0–S1 vs. S2–S3	≥18.52	63.5 (57.3–69.7)	95.1 (92.7–97.1)	87.7 (82.5–92.5)	82.7 (80.3–85.2)
S0–S2 vs. S3	≥25.15	50.5 (41.0–61.0)	95.0 (93.0–96.8)	65.8 (56.2–75.8)	91.1 (89.4–92.8)
**Sensitivity 90% (rule-out thresholds)**					
S0 vs. S1–S3	<4.11	90.2 (87.6–92.8)	81.2 (74.6–87.7)	94.1 (92.2–96.1)	71.4 (65.6–77.5)
S0–S1 vs. S2–S3	<11.49	90.0 (85.8–93.8)	81.3 (77.5–85.5)	72.5 (68.6–77.1)	93.7 (91.3–96.1)
S0–S2 vs. S3	<15.06	90.5 (84.2–95.8)	73.7 (69.9–77.5)	39.5 (36.0–43.4)	97.6 (96.1–99.0)
**Sensitivity 95% (rule-out thresholds)**					
S0 vs. S1–S3	<2.53	95.2 (93.0–96.9)	53.6 (45.6–61.6)	87.2 (85.3–89.2)	77.4 (69.2–84.8)
S0–S1 vs. S2–S3	<10.17	95.3 (92.4–97.6)	75.6 (71.2–80.0)	68.1 (64.3–72.4)	96.7 (94.6–98.4)
S0–S2 vs. S3	<11.39	95.8 (91.6–98.9)	65.7 (61.3–70.1)	34.6 (31.7–37.9)	98.8 (97.6–99.7)

Abbreviations: Se, sensitivity; Sp, specificity; PPV, positive predictive value; NPV, negative predictive value; CI, confidence interval.

Supplementary Figures

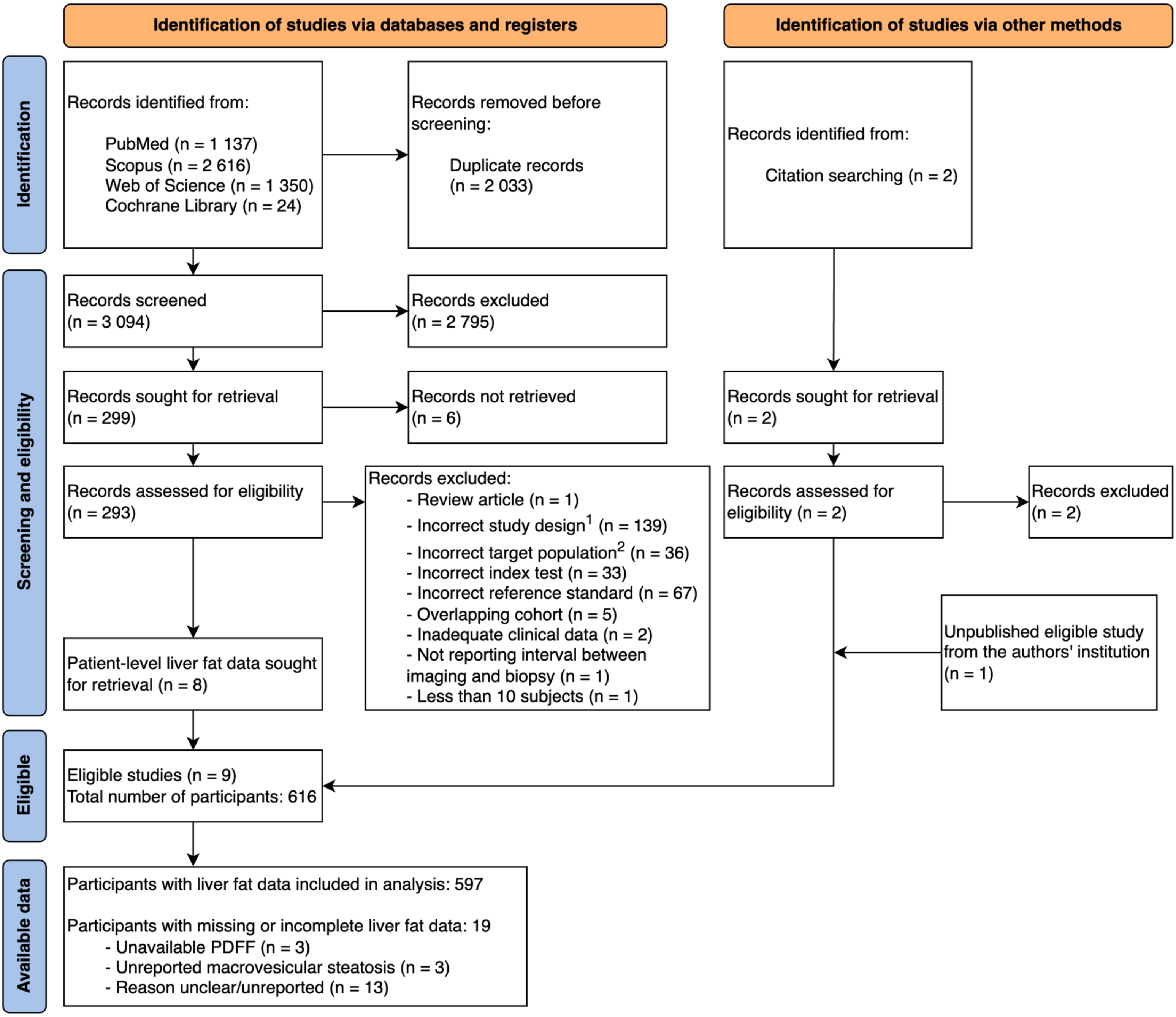

[1]Including studies not reporting data on associations between histological steatosis and PDFF.

[2]Including studies conducted in pediatric populations, with animals, or *ex vivo*, as well as studies including patients with primary liver diseases other than NAFLD, with liver cancer, or with metastases.

Adopted from: Page MJ, McKenzie JE, Bossuyt PM, Boutron I, Hoffmann TC, Mulrow CD, et al. The PRISMA 2020 statement: an updated guideline for reporting systematic reviews. BMJ 2021;372:n71. doi: 10.1136/bmj.n71. For more information, visit: http://www.prisma-statement.org/

Fig. S1. PRISMA flow diagram for the study selection.

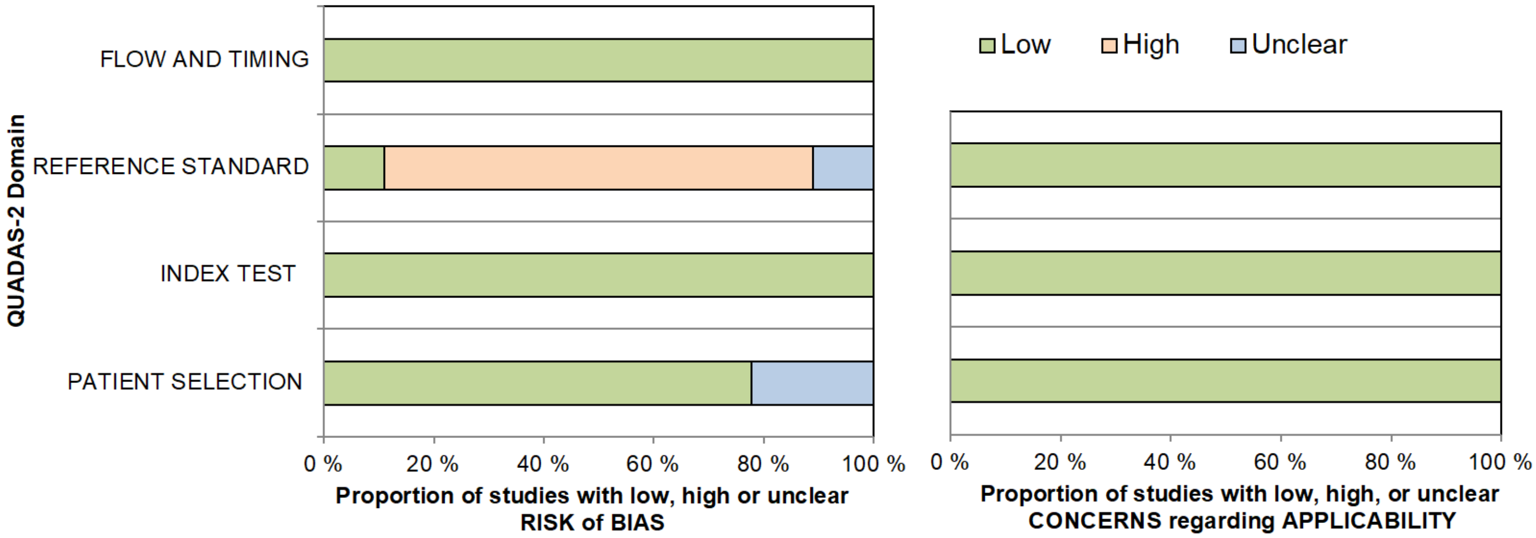

Fig. S2. QUADAS-2 quality and risk-of-bias assessment.

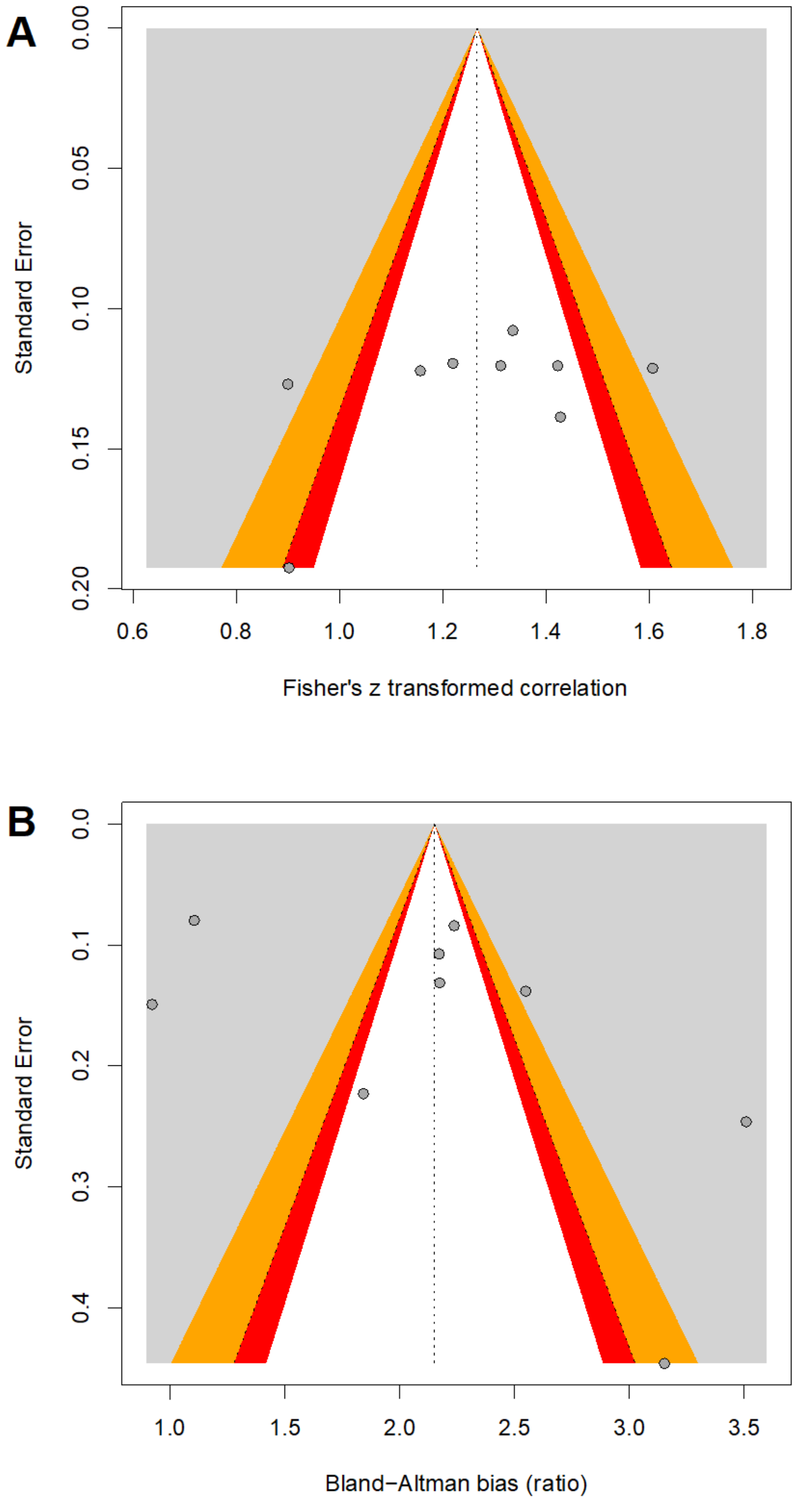

Fig. S3. Contour-enhanced funnel plots showing distribution of the included studies with respect to their (A) Fisher's z transformed Pearson correlation coefficients and their standard errors and (B) proportional Bland-Altman bias estimates and their standard errors. Red zones denote 90–95% confidence limits, while orange zones denote 95–99% confidence limits.

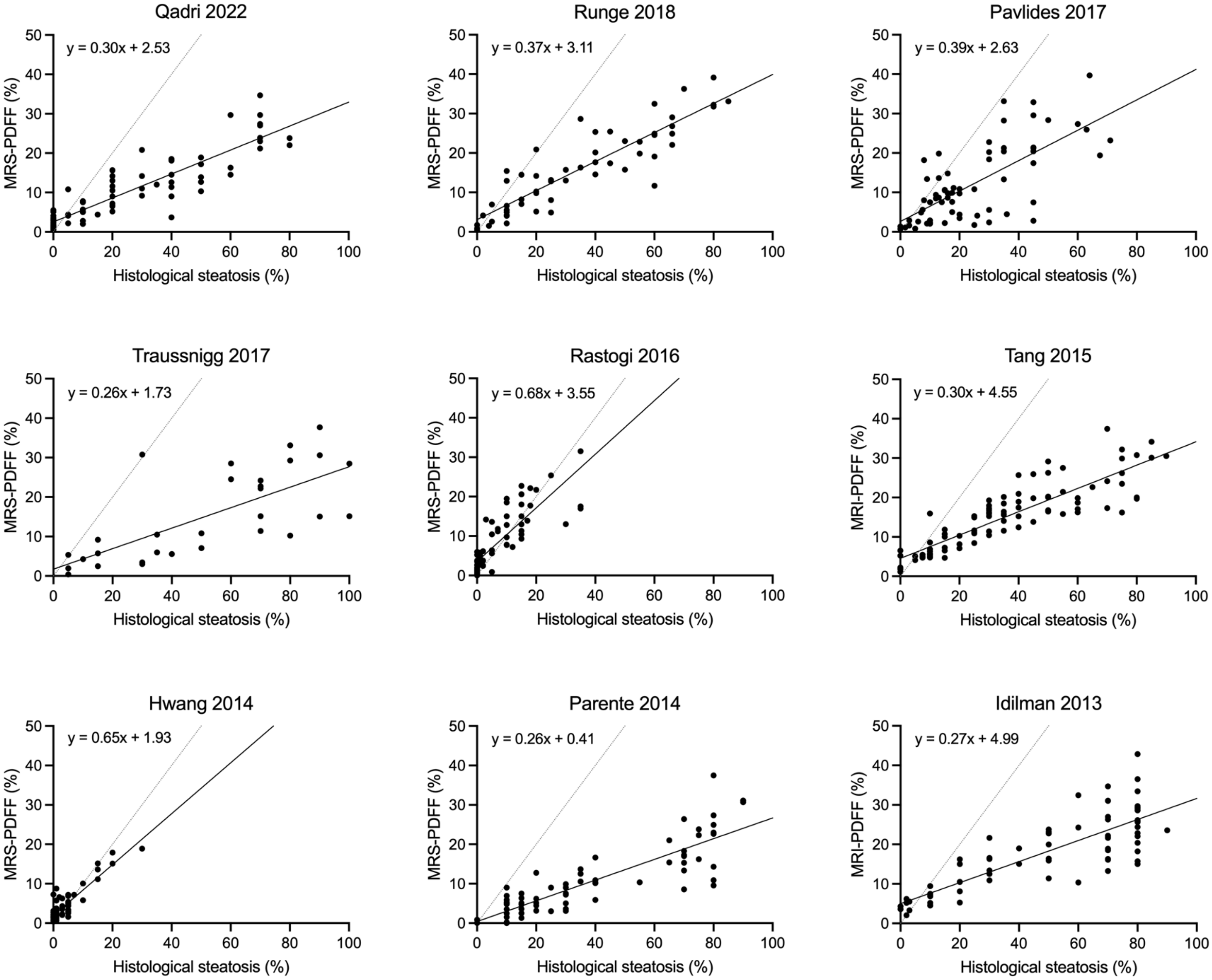

Fig. S4. Relationship between histological steatosis and PDFF in the individual studies. Lines were fit using linear regression. Dotted gray line is the line of identity.

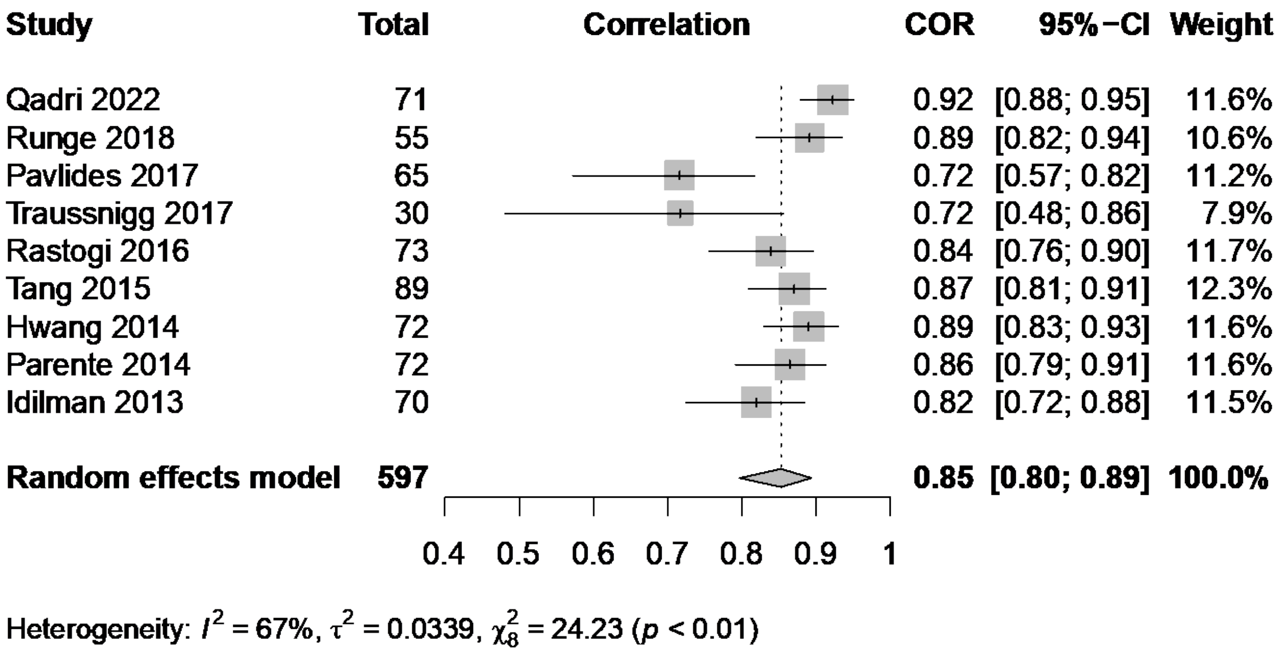

Fig. S5. Random-effects meta-analysis of Pearson correlation coefficients for histological steatosis and PDFF in the included studies.

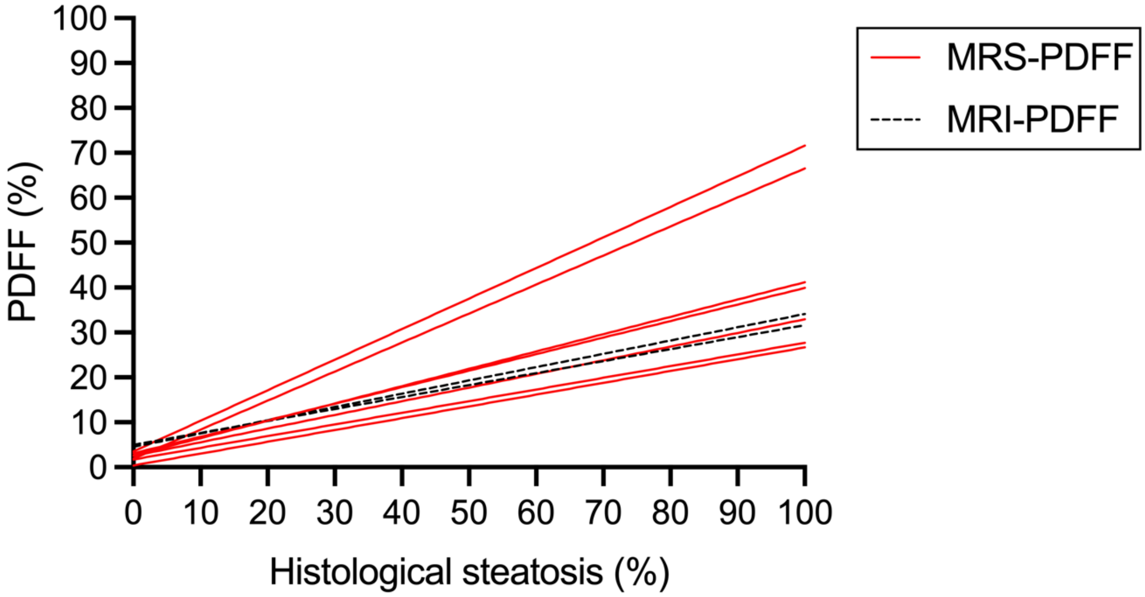

Fig. S6. Linear regression lines for the individual studies showing associations between histological steatosis and PDFF. Solid red lines denote studies using MRS-PDFF, and dashed black lines denote studies using MRI-PDFF.

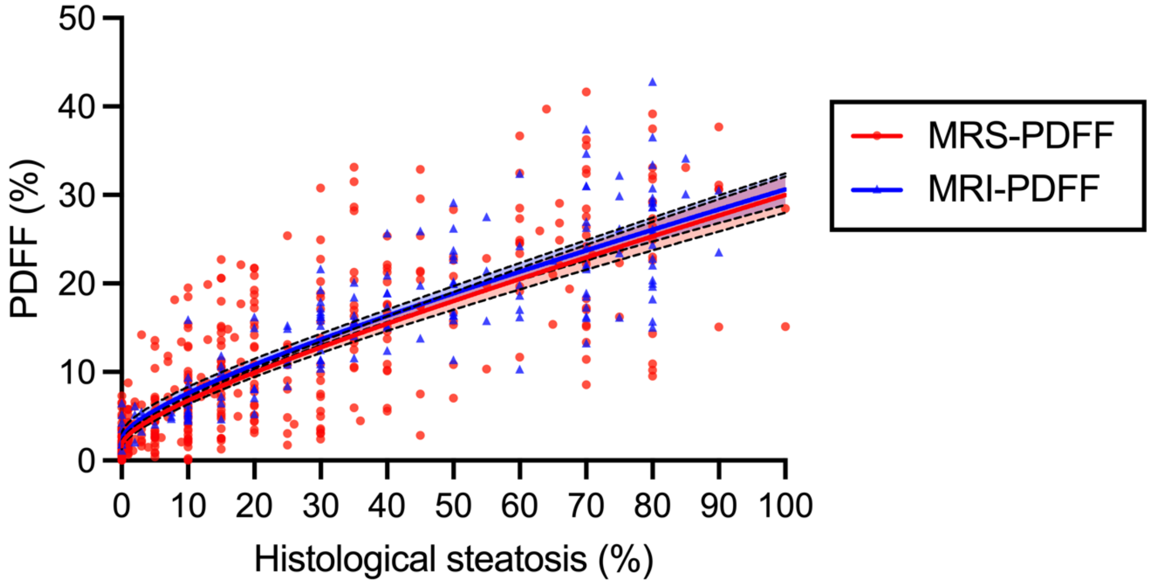

Fig. S7. Relationship between histological steatosis and PDFF, stratified by use of either MRS-PDFF or MRI-PDFF. The solid red circles and the red line denote MRS-PDFF, and the solid blue triangles and the blue line denote MRI-PDFF. The best-fit lines were determined using linear regression. Both variables underwent square root transformation prior to model fitting, and the curve fit was then backtransformed for display. The shaded areas denote 95% CI.

Supplementary References

[1] Bedossa P, Poitou C, Veyrie N, Bouillot JL, Basdevant A, Paradis V, et al. Histopathological algorithm and scoring system for evaluation of liver lesions in morbidly obese patients. Hepatology 2012;56:1751-1759.

[2] Bedossa P, FLIP Pathology Consortium. Utility and appropriateness of the fatty liver inhibition of progression (FLIP) algorithm and steatosis, activity, and fibrosis (SAF) score in the evaluation of biopsies of nonalcoholic fatty liver disease. Hepatology 2014;60:565-575.

[3] Vanhamme L, van den Boogaart A, Van Huffel S. Improved method for accurate and efficient quantification of MRS data with use of prior knowledge. Journal of magnetic resonance 1997;129:35-43.

[4] The Genotype-Tissue Expression (GTEx) Consortium. GTEx V8 Analysis Methods. Updated 2019 Aug 20 [cited 2022 Sept 12]; Available from: https://gtexportal.org/home/methods

[5] Dobin A, Davis CA, Schlesinger F, Drenkow J, Zaleski C, Jha S, et al. STAR: ultrafast universal RNA-seq aligner. Bioinformatics 2013;29:15-21.

[6] DeLuca DS, Levin JZ, Sivachenko A, Fennell T, Nazaire MD, Williams C, et al. RNA-SeQC: RNA-seq metrics for quality control and process optimization. Bioinformatics 2012;28:1530-1532.

[7] Newman AM, Steen CB, Liu CL, Gentles AJ, Chaudhuri AA, Scherer F, et al. Determining cell type abundance and expression from bulk tissues with digital cytometry. Nat Biotechnol 2019;37:773-782.

[8] MacParland SA, Liu JC, Ma XZ, Innes BT, Bartczak AM, Gage BK, et al. Single cell RNA sequencing of human liver reveals distinct intrahepatic macrophage populations. Nat Commun 2018;9:4383.

[9] Runge JH, Smits LP, Verheij J, Depla A, Kuiken SD, Baak BC, et al. MR Spectroscopy-derived Proton Density Fat Fraction Is Superior to Controlled Attenuation Parameter for Detecting and Grading Hepatic Steatosis. Radiology 2018;286:547-556.

[10] Pavlides M, Banerjee R, Tunnicliffe EM, Kelly C, Collier J, Wang LM, et al. Multiparametric magnetic resonance imaging for the assessment of non-alcoholic fatty liver disease severity. Liver Int 2017;37:1065-1073.

[11] Traussnigg S, Kienbacher C, Gajdosik M, Valkovic L, Halilbasic E, Stift J, et al. Ultra-high-field magnetic resonance spectroscopy in non-alcoholic fatty liver disease: Novel mechanistic and diagnostic insights of energy metabolism in non-alcoholic steatohepatitis and advanced fibrosis. Liver Int 2017;37:1544-1553.

[12] Rastogi R, Gupta S, Garg B, Vohra S, Wadhawan M, Rastogi H. Comparative accuracy of CT, dual-echo MRI and MR spectroscopy for preoperative liver fat quantification in living related liver donors. Indian J Radiol Imaging 2016;26:5-14.

[13] Tang A, Desai A, Hamilton G, Wolfson T, Gamst A, Lam J, et al. Accuracy of MR imaging-estimated proton density fat fraction for classification of dichotomized histologic steatosis grades in nonalcoholic fatty liver disease. Radiology 2015;274:416-425.

[14] Hwang I, Lee JM, Lee KB, Yoon JH, Kiefer B, Han JK, Choi BI. Hepatic steatosis in living liver donor candidates: preoperative assessment by using breath-hold triple-echo MR imaging and 1H MR spectroscopy. Radiology 2014;271:730-738.

[15] Parente DB, Rodrigues RS, Paiva FF, Oliveira Neto JA, Machado-Silva L, Lanzoni V, et al. Is MR spectroscopy really the best MR-based method for the evaluation of fatty liver in diabetic patients in clinical practice? PLoS One 2014;9:e112574.

[16] Idilman IS, Aniktar H, Idilman R, Kabacam G, Savas B, Elhan A, et al. Hepatic steatosis: quantification by proton density fat fraction with MR imaging versus liver biopsy. Radiology 2013;267:767-775.
