# Supplementary material for: Marked difference in liver fat measured by histology vs. magnetic resonance-proton density fat fraction: A meta-analysis
Source: JHEP Rep. 2023 Oct 11;6(1):100928. doi: 10.1016/j.jhepr.2023.100928 (PMC10711480; doi:10.1016/j.jhepr.2023.100928)
Supplement: Multimedia component 2 — : [file mmc2.docx]

**JHEP Reports**

**CTAT methods**

Tables for a “Complete, Transparent, Accurate and Timely account” (CTAT) are now mandatory for all revised submissions. The aim is to enhance the reproducibility of methods.

- Only include the parts relevant to your study
- Refer to the CTAT in the main text as ‘Supplementary CTAT Table’
- Do not add subheadings
- Add as many rows as needed to include all information
- Only include one item per row

**If the CTAT form is not relevant to your study, please outline the reasons why:**

|  |
| --- |

- 1. **Antibodies**

| **Name** | **Citation** | **Supplier** | **Cat no.** | **Clone no.** |
| --- | --- | --- | --- | --- |
| N/A | N/A | N/A | N/A | N/A |

- 1. **Cell lines**

| **Name** | **Citation** | **Supplier** | **Cat no.** | **Passage no.** | **Authentication test method** |
| --- | --- | --- | --- | --- | --- |
| N/A | N/A | N/A | N/A | N/A | N/A |

- 1. **Organisms**

| **Name** | **Citation** | **Supplier** | **Strain** | **Sex** | **Age** | **Overall n number** |
| --- | --- | --- | --- | --- | --- | --- |
| N/A | N/A | N/A | N/A | N/A | N/A | N/A |

- 1. **Sequence based reagents**

| **Name** | **Sequence** | **Supplier** |
| --- | --- | --- |
| N/A | N/A | N/A |

- 1. **Biological samples**

| **Description** | **Source** | **Identifier** |
| --- | --- | --- |
| Human liver biopsies | Helsinki Univ. Hospital | N/A |

- 1. **Deposited data**

| **Name of repository** | **Identifier** | **Link** |
| --- | --- | --- |
| N/A | N/A | N/A |

- 1. **Software**

| **Software name** | **Manufacturer** | **Version** |
| --- | --- | --- |
| GraphPad Prism | GraphPad Software, La Jolla, CA | 9.3.1 |
| R | R Foundation for Statistical Computing, Vienna, Austria | 4.1.2 |
| EndNote | Clarivate, Philadelphia, PA | 20.2 |
| Rayyan | Rayyan Systems Inc., Cambridge, MA, | N/A |

- 1. **Other (*e.g*. drugs, proteins, vectors etc.)**

| N/A | N/A | N/A |
| --- | --- | --- |

- 1. **Please provide the details of the corresponding methods author for the manuscript:**

| Sami Qadri, MD  Department of Medicine, University of Helsinki  Biomedicum Helsinki 1, room A418a  Haartmaninkatu 8, 00290 Helsinki, Finland  E-mail: sami.qadri@helsinki.fi |
| --- |

**2.0 Please confirm for randomised controlled trials all versions of the clinical protocol are included in the submission. These will be published online as supplementary information.**

| N/A |
| --- |
