# Supplementary material for: Marked difference in liver fat measured by histology vs. magnetic resonance-proton density fat fraction: A meta-analysis
Source: JHEP Rep. 2023 Oct 11;6(1):100928. doi: 10.1016/j.jhepr.2023.100928 (PMC10711480; doi:10.1016/j.jhepr.2023.100928)
Supplement: Multimedia component 4 [file mmc4.pdf]

Research article JHEP|Reports

Marked difference in liver fat measured by histology *vs.* magnetic resonance-proton density fat fraction: A meta-analysis

Authors

Sami Qadri, Emilia Vartiainen, Mari Lahelma, Kimmo Porthan, An Tang, Ilkay S. Idilman, Jurgen H. Runge, Anne Juuti, Anne K. Penttilä, Juhani Dabek, Tiina E. Lehtimäki, Wenla Seppänen, Johanna Arola, Perttu Arkkila, Jaap Stoker, Musturay Karcaaltincaba, Michael Pavlides, Rohit Loomba, Claude B. Sirlin, Taru Tukiainen, Hannele Yki-Järvinen.

Correspondence

sami.qadri@helsinki.fi (S. Qadri), hannele.yki-jarvinen@helsinki.fi (H. Yki-Järvinen).

Graphical abstract

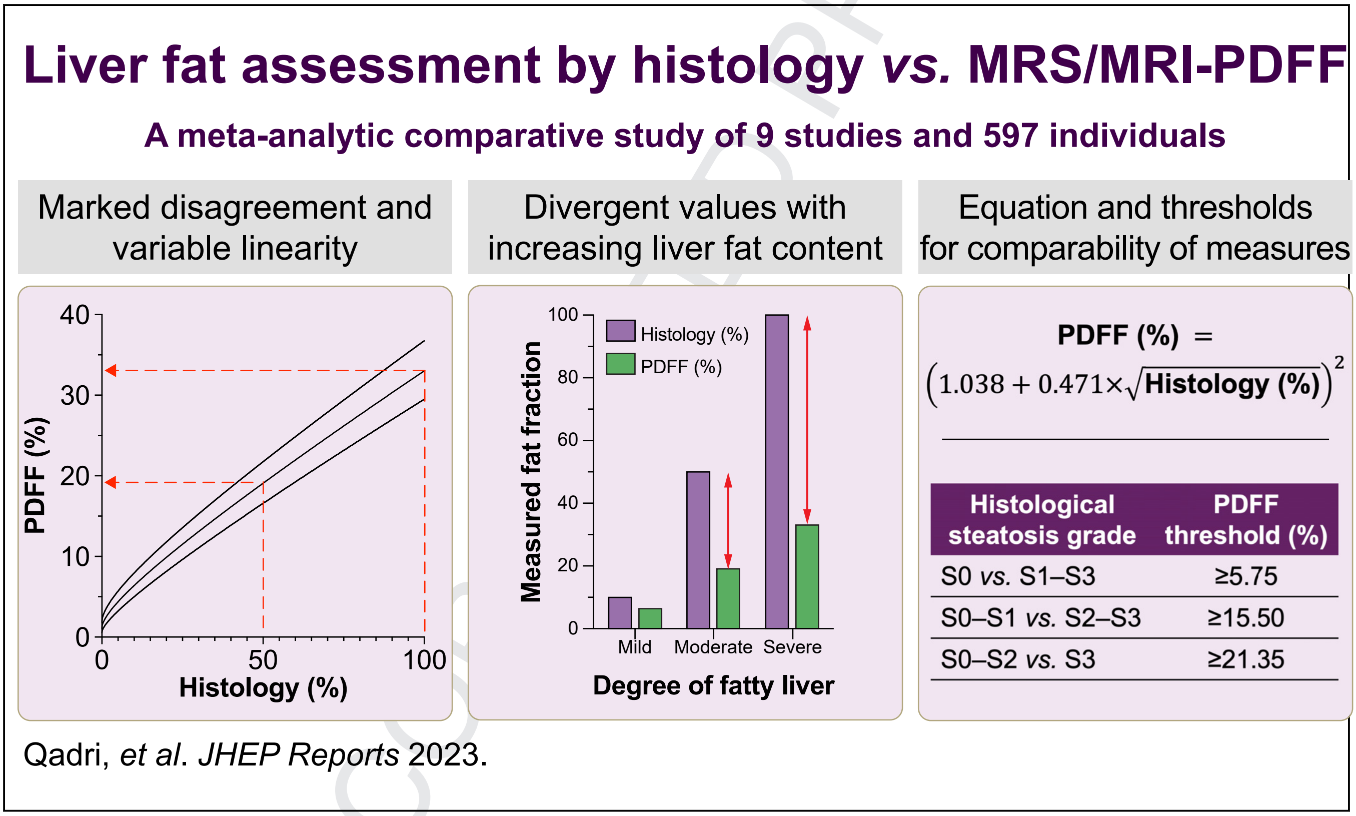

Highlights

- Histology and PDFF are fundamentally different methods of liver fat quantification.
- The relationship between histological steatosis and PDFF is predominantly linear.
- Percentage liver fat by histology is often markedly higher compared to PDFF.
- Differences between histological steatosis and PDFF increase with higher liver fat.
- A formula or threshold values enable comparison of histological steatosis and PDFF.

Impact and implications

In patients with fatty liver disease, the amount of liver fat can be measured by microscopic analysis of a liver biopsy or non-invasively by magnetic resonance. We assessed whether these two methods, both reporting liver fat as percentage values, yield comparable results. Liver biopsy analysis consistently produced higher liver fat values compared with magnetic resonance, and differences between the methods markedly increased as a function of fatty liver severity. Measurements obtained using the two methods may be rendered comparable by conversion equations and lookup tables. Clinical practitioners should be aware that liver biopsy and magnetic resonance represent fundamentally different methods of liver fat assessment and that their results should be interpreted independent of each other.

https://doi.org/10.1016/j.jhepr.2023.100928

UNCORRECTED PROOF

UNCORRECTED PROOF

Q1Q12

Marked difference in liver fat measured by histology *vs.* magnetic resonance-proton density fat fraction: A meta-analysis

Q11 Sami Qadri,[1,2,*,†] Emilia Vartiainen,[3] Mari Lahelma,[1,2] Kimmo Porthan,[1,2] An Tang,[4] Ilkay S. Idilman,[5] Jurgen H. Runge,[6,7] Anne Juuti,[8] Anne K. Penttilä,[8] Juhani Dabek,[1,2] Tiina E. Lehtimäki,[9] Wenla Seppänen,[9] Johanna Arola,[10] Perttu Arkkila,[11] Jaap Stoker,[6,7] Musturay Karcaaltincaba,[5] Michael Pavlides,[12] Rohit Loomba,[13] Claude B. Sirlin,[14] Taru Tukiainen,[3] Hannele Yki-Järvinen[1,2,*,†]

[1]Department of Medicine, University of Helsinki and Helsinki University Hospital, Helsinki, Finland; [2]Minerva Foundation Institute for Medical Research, Helsinki, Finland; [3]Institute for Molecular Medicine Finland, FIMM, University of Helsinki, Helsinki, Finland; [4]Department of Radiology, Centre hospitalier de l'Université de Montréal (CHUM), Montreal, QC, Canada; [5]Liver Imaging Team, Hacettepe University, School of Medicine, Department of Radiology, Ankara, Turkey; [6]Department of Radiology and Nuclear Medicine, Amsterdam UMC location University of Amsterdam, Amsterdam, The Netherlands; [7]Amsterdam Gastroenterology Endocrinology Metabolism, Amsterdam, The Netherlands; [8]Department of Gastrointestinal Surgery, Abdominal Center, University of Helsinki and Helsinki University Hospital, Helsinki, Finland; [9]HUS Medical Imaging Center, Helsinki University Hospital, Helsinki, Finland; [10]Department of Pathology, University of Helsinki and Helsinki University Hospital, Helsinki, Finland; [11]Department of Gastroenterology, Abdominal Center, Helsinki University Hospital and University of Helsinki, Helsinki, Finland; [12]Radcliffe Department of Medicine, University of Oxford, Oxford, UK; [13]NAFLD Research Center, Division of Gastroenterology and Hepatology, Department of Medicine, University of California San Diego, La Jolla, CA, USA; [14]Liver Imaging Group, Department of Radiology, University of California San Diego, La Jolla, CA, USA

JHEP Reports **2023.** https://doi.org/10.1016/j.jhepr.2023.100928

Q2 **Background & Aims:** Pathologists quantify steatosis as the fraction of lipid droplet-containing hepatocytes out of all hepatocytes, whereas the magnetic resonance-determined proton density fat fraction (PDFF) reflects the tissue triacylglycerol concentration. We investigated the linearity, agreement, and correspondence thresholds between histological steatosis and PDFF across the full clinical spectrum of liver fat content associated with non-alcoholic fatty liver disease.
Methods: Using individual patient-level measurements, we conducted a systematic review and meta-analysis of studies comparing histological steatosis with PDFF determined by magnetic resonance spectroscopy or imaging in adults with suspected non-alcoholic fatty liver disease. Linearity was assessed by meta-analysis of correlation coefficients and by linear mixed modelling, agreement by Bland–Altman analysis, and thresholds by receiver operating characteristic analysis. To explain observed differences between the methods, we used RNA-seq to determine the fraction of hepatocytes in human liver biopsies.
Results: Eligible studies numbered 9 (N = 597). The relationship between PDFF and histology was predominantly linear (r = 0.85 [95% CI, 0.80–0.89]), and their values approximately coincided at 5% steatosis. Above 5% and towards higher levels of steatosis, absolute values of the methods diverged markedly, with histology exceeding PDFF by up to 3.4-fold. On average, 100% histological steatosis corresponded to a PDFF of 33.0% (29.5–36.7%). Targeting at a specificity of 90%, optimal PDFF thresholds to predict histological steatosis grades were ≥5.75% for ≥S1, ≥15.50% for ≥S2, and ≥21.35% for S3. Hepatocytes comprised 58 ± 5% of liver cells, which may partly explain the lower values of PDFF *vs.* histology.
Conclusions: Histological steatosis and PDFF have non-perfect linearity and fundamentally different scales of measurement. They require independent interpretation to prevent misjudgement of the clinical status or treatment effect in patient care.
Impact and implications: In patients with fatty liver disease, the amount of liver fat can be measured by microscopic analysis of a liver biopsy or non-invasively by magnetic resonance. We assessed whether these two methods, both reporting liver fat as percentage values, yield comparable results. Liver biopsy analysis consistently produced higher liver fat values compared with magnetic resonance, and differences between the methods markedly increased as a function of fatty liver severity. Measurements obtained using the two methods may be rendered comparable by conversion equations and lookup tables. Clinical practitioners should be aware that liver biopsy and magnetic resonance represent fundamentally different methods of liver fat assessment and that their results should be interpreted independent of each other.
© 2023 The Author(s). Published by Elsevier B.V. on behalf of European Association for the Study of the Liver (EASL). This is an open access article under the CC BY license (http://creativecommons.org/licenses/by/4.0/).

UNCORRECTED PROOF

Keywords: Fatty liver; Metabolic dysfunction-associated steatotic liver disease; Magnetic resonance imaging; Magnetic resonance spectroscopy; Biopsy; Histology; Hepatocytes; Pathologists; Triglycerides; Transcriptome; Systematic review; Meta-analysis.
Received 19 April 2023; received in revised form 17 August 2023; accepted 12 September 2023; available online XXX
† Co-corresponding authors.
* Corresponding authors. Address: Biomedicum Helsinki 1, Room A418a, Haartmaninkatu 8, 00290 Helsinki, Finland. Tel: +358-50-562-6899..
E-mail addresses: sami.qadri@helsinki.fi (S. Qadri), hannele.yki-jarvinen@helsinki.fi (H. Yki-Järvinen).

Introduction

In histological evaluation of liver fat, the pathologist visually estimates the fraction of lobular hepatocytes containing macrovesicular lipid droplets.[1] To diagnose non-alcoholic fatty liver disease (NAFLD), the recommended steatosis cut-off in American,[2] European,[3] and Asian-Pacific guidelines,[4] as well as in textbooks,[5] is 5%. Although pathologist scoring is generally the most concordant for macrovesicular steatosis as compared with other features of NAFLD, it is nevertheless subject to significant inter-rater variability and often graded using a four-point scale ranging from S0 to S3 (S0: <5%; S1: 5–33%; S2: 34–66%; S3: >66%).[1]

In lieu of histology, magnetic resonance (MR)-based techniques are increasingly used to measure liver fat accurately and non-invasively.[6,7] Within this domain, *in vivo* proton MR spectroscopy (^{1}H-MRS, later MRS) is the reference standard, as it enables direct calculation of the tissue proton density fat fraction (PDFF) from signal intensities of spectral peaks originating from mobile protons in hepatic triacylglycerols and water.[7] However, as MRS requires specialised equipment and expertise to both acquire and analyse spectral data, it has in part been superseded by MR imaging (MRI)-based indirect quantification of PDFF.[6] A recent meta-analysis with 23 studies and 1,679 patients showed MRS-PDFF and MRI-PDFF to be essentially in complete agreement, with an R^2 of 0.96 between the modalities.[8]

In subjects of the Dallas Heart Study without a liver biopsy, the upper limit of normal for liver fat by MRS-PDFF was considered 5.56%—a cut-off closely approximating the histological definition of NAFLD.[9] However, the exact relationship between PDFF and histologically determined steatosis fraction remains enigmatic. Although there generally exists a high correlation between PDFF and histology, use of crude scoring systems instead of more granular pathologist-reported steatosis fractions in most comparative studies has obscured their numerical relationship.[10–15] Importantly, the theoretical basis of the methods suggests them to be fundamentally different. PDFF measures the volumetric tissue concentration of triacylglycerol, calculated as the ratio of MR-visible triacylglycerol protons to the sum of protons in triacylglycerol and water.[7] However, pathologists estimate on the proportion of hepatocytes containing macrovesicular lipid droplets, out of all hepatocytes within a histological cross-section. Although previous authors have acknowledged these differences, the likely effect on the methods' concordance has not been systematically examined.[7,16–19] Additionally, as MRS and MRI probe the liver without discriminating signal from different cell types, the sole consideration of hepatocytes by pathologists may act as an additional confounder. To the best of our knowledge, the proportion of hepatocytes out of all cells in human liver tissue remains undetermined.

With the increasing popularity of PDFF, knowledge by clinicians as to how it corresponds to histological steatosis fraction is important to prevent misjudgement of the clinical status or treatment effect in patient care. However, most guidelines and expert recommendations on non-invasive assessment of NAFLD have failed to acknowledge the potential differences between these key methods of steatosis assessment.[2,3,20] This may be because of the lack of studies formally comparing their characteristics in sufficiently large populations.

Our aim was to determine the degree of linearity and agreement between histological steatosis fraction and PDFF, across the full clinical spectrum of liver fat content associated with NAFLD. To this end, we performed a systematic review with meta-analytic assessment of patient-level data, including unpublished data from our institution. Because we found the methods to be in considerable disagreement, we derived a general equation and correspondence thresholds for relating PDFF with histological results. Finally, to explore the significance of the non-parenchymal hepatic cell fraction as a confounder of steatosis measurement, we determined the cell-type composition of human liver biopsies.

Materials and methods

Systematic review of the literature

Two investigators (SQ and HYJ) independently conducted a literature search to identify peer-reviewed articles and meeting abstracts of any language reporting associations between the pathologist-reported histological macrovesicular steatosis fraction and PDFF. We considered studies using either MRS or confounder-corrected chemical shift-encoded MRI, as the methods provide essentially identical measures of PDFF.[8] Expert recommendations for appropriate confounder correction in PDFF acquisition have been published elsewhere.[7] The target population was adults undergoing a liver biopsy either because of suspected NAFLD or in conjunction with routine work-up of living liver donor candidates, with the exclusion of other primary liver diseases (see below). We followed the Preferred Reporting Items for Systematic Reviews and Meta-analyses (PRISMA) reporting guidelines.[21] An institutional review board approval was not required for this systematic review. The review protocol was not publicly registered.

Search strategy

The literature search consisted of three main concepts: (1) liver fat or fatty liver disease; (2) biopsy or histology; and (3) MRI or MRS.

The MEDLINE (via PubMed), CENTRAL (via the Cochrane Library), Embase (via Scopus), and Web of Science Core Collection databases were searched from database inception until 16 August 2022. The search was initially built in PubMed and was subsequently translated to other databases as accurately as possible. Controlled vocabulary was used where appropriate, supplemented with (truncated) keywords. A detailed electronic search strategy is provided in Table S1.

Identification of eligible studies

Search results were exported from each database and imported to EndNote version 20.2 (Clarivate, Philadelphia, PA, USA) for deduplication. The deduplicated reference library was then exported from EndNote to the Rayyan web application (Rayyan Systems Inc., Cambridge, MA, USA) for screening of titles and abstracts for potential eligibility by the lead author (SQ).[22] Bibliographic data of the potentially eligible studies were again imported to EndNote for reviewing of full-text records. After identification of all the eligible studies, their reference lists were reviewed to identify additional reports for inclusion. Additionally, relevant systematic reviews and meta-analyses were tagged for subsequent review of their reference lists to identify additional reports.

Study selection

Studies were selected if they fulfilled the following inclusion criteria:

ARTICLE IN PRESS

(1) Study design: any controlled trials, comparative studies, and observational studies.
(2) Target population: adults undergoing a liver biopsy because of suspected NAFLD or during work-up as living liver donor candidates.
(3) Reference standard: a pathologist's assessment of histological steatosis fraction in liver biopsies, defined as the fraction of lobular hepatocytes containing macrovesicular lipid droplets out of all hepatocytes.
(4) Index tests: liver fat content measured by MRS/MRI-PDFF within 180 days (on average) of undergoing liver biopsy.

In addition, the following exclusion criteria were exercised:

(1) Not reporting data on associations between histological steatosis and PDFF.
(2) Studies conducted in paediatric populations, with animals, or *ex vivo*.
(3) Studies including fewer than 10 subjects.
(4) Studies including patients with primary liver diseases other than NAFLD or with liver cancer or metastases, and studies with insufficient reporting to ascertain correct target population. Studies including patients with other primary liver diseases were considered if data for patients with NAFLD could be extracted separately.
(5) Ordinal reference standard (*i.e.* steatosis grade instead of macrovesicular steatosis fraction) or incorrect index test, or insufficient reporting to ascertain eligibility.
(6) Insufficient characterisation of the study population (at least the number of males/females, mean age, and mean BMI should be reported).

Data extraction

The lead author (SQ) extracted the following study-level data: author, year, country, study design, and index test. Regarding patient-level data, we extracted information about the target population, number of participants, sex distribution, mean age, mean BMI, histological diagnoses, and the average interval between imaging and biopsy. Additionally, we extracted the following information regarding the index test: scanner manufacturer, field strength, repetition time, echo time, number of echoes, number of voxels/regions of interest, dimensionality (for MRI), reconstruction method (for MRI), and pulse sequence (for MRS).

A requirement for study inclusion was access to individual patient-level data for histological and MR-based liver fat measurements. Corresponding authors of the selected studies were contacted by e-mail to request raw data for this meta-analysis, and the authors were given 60 days to respond. If no response was received within this timeframe, we digitised the data from published figures.

Quality and risk-of-bias assessment

We assessed the methodological quality and risk-of-bias of the included studies using the QUADAS-2 tool.[23] With QUADAS-2, methodological quality is assessed across four domains: (1) patient selection; (2) index test; (3) reference standard; and (4) flow and timing. The tool was appropriately tailored for use in this systematic review. Because of the known poor inter-rater agreement in macrovesicular steatosis assessment,[24] risk-of-bias for the reference standard was deemed high unless the study utilised a consensus reading of at least two pathologists.

The Helsinki University Hospital MRS-PDFF cohort

In the present meta-analysis, we included unpublished data from 71 eligible individuals who were studied at our institution. Detailed methodology regarding the Helsinki MRS-PDFF cohort is described in the Supplementary material, and clinical characteristics are shown in Table S2.

Hepatic cell-type composition analysis

To determine the fractional contributions of different cell types in human liver tissue, we used an RNA-seq-based computational approach (CIBERSORTx) and a previously published human liver single-cell RNA-seq dataset in a liver biopsy cohort consisting of 138 patients.[25,26] The methods are described in the Supplementary material, and characteristics of the cohort are shown in Table S3.

Statistical methods

Analyses were performed using R version 4.1.2 (R Foundation for Statistical Computing, Vienna, Austria) or GraphPad Prism version 9.3.1 (GraphPad Software, San Diego, CA, USA) for macOS. The R package 'meta' version 5.2-0 was used to derive all meta-analytic estimates,[27] and the package 'lme4' version 1.1-28 was used for mixed-effects modelling.[28] Data are shown as means ± standard deviations, medians (25th–75th percentiles), or counts (percentages).

Evaluation of publication bias

We assessed the possibility of underlying publication bias and other small-study effects by using funnel plots. Effect estimates included Fisher's z transformed Pearson correlation coefficients and their standard errors (the main measure of linearity), and proportional Bland–Altman bias estimates and their standard errors (the main measure of agreement). We evaluated funnel plot asymmetry with the Egger's test, using $p \leq 0.05$ as a threshold for statistically significant asymmetry.

Linearity between histological steatosis and PDFF

Using a two-stage approach, Pearson correlation coefficients derived for each individual study underwent meta-analytic assessment after Fisher's z transformation using a random-effects model and inverse variance weighting. Test statistics and confidence intervals were adjusted by using the method of Hartung and Knapp.

Agreement between histological steatosis and PDFF

Agreement was assessed using a one-stage approach. Because of a non-constant relationship between the measures, non-linear regression was used to fit lines in Bland–Altman plots describing bias over the full range of liver fat content. To describe the average relationship between histological steatosis and PDFF, a linear mixed model was fit in the pooled dataset. Heteroscedasticity and non-normality of residuals was rectified via square root transformation of the variables. The curve fit was then back-transformed for display. Study effects were considered as random effects in all analyses.

Classifying histological steatosis grades by PDFF

We used receiver operating characteristic (ROC) analysis and area under the ROC curve (AUROC) for studying the

FLA 5.6.0 DTD ■ JHEPR100928_proof ■ 15 November 2023 ■ 10:25 pm ■ ce

UNCORRECTED PROOF

Table 1. Characteristics of the included studies.

Author, year, country, ref.	Index method	Study design	Target population	Number of participants (m/f)	Patient demographics	Histological diagnosis	Interval between imaging and biopsy
Qadri, 2022, Finland*	MRS-PDFF	Prospective	Patients undergoing liver biopsy to evaluate NAFLD during metabolic surgery	21/50	Age: 52 ± 11 yr<br>BMI: 37.6 [32.9, 41.2] kg/m^2	No NAFLD: 23<br>NAFL: 29<br>NASH: 19	7.2 [2.8, 15.7] d
Runge, 2018, The Netherlands[29]	MRS-PDFF	Prospective	Patients undergoing liver biopsy due to suspected NAFLD	40/15	Age: 52.3 [43.7, 57.6] yr<br>BMI: 27.8 [26.0, 33.1] kg/m^2	No NAFLD: 5<br>NAFL: 30<br>NASH: 20	27 [7, 44] d
Pavlides, 2017, UK[30]	MRS-PDFF	Prospective	Patients with known or suspected NAFLD undergoing liver biopsy	43/28 (65)†	Age: 53 ± 12 yr<br>BMI: 32.7 [28.1, 38.1] kg/m^2	NAFL: 25<br>NASH: 46	13 [5, 27] d
Traussnigg, 2017, Austria[31]	MRS-PDFF	Prospective	Patients undergoing liver biopsy because of suspected NAFLD	18/12	Patients with NAFL:<br>Age: 48.0 ± 9.6 yr<br>BMI: 27.3 ± 5.2 kg/m^2<br>Patients with NASH:<br>Age 48.0 ± 12.5 yr<br>BMI 31.4 ± 4.1 kg/m^2	NAFL: 8<br>NASH: 22	Performed on the same day
Rastogi, 2016, India[32]	MRS-PDFF	Retrospective	Living liver donor candidates undergoing preoperative or intraoperative liver biopsy	59/14	Males:<br>Age: 33 (20–55) yr<br>BMI: 24.6 (17.2–34.8) kg/m^2<br>Females:<br>Age: 33 (19–55) yr<br>BMI: 24.7 (17.9–29.8) kg/m^2	No NAFLD: 39<br>NAFL: 34	≤20 d
Tang, 2015, USA[33]	MRI-PDFF	Prospective	Patients with known or suspected NAFLD undergoing liver biopsy	38/51	Age: 51.0 ± 13.0 yr<br>BMI: 30.6 ± 5.0 kg/m^2	No NAFLD: 6<br>NAFLD: 83	Median 35 (range 0–173) d
Hwang, 2014, Republic of Korea[34]	MRS-PDFF	Retrospective	Living liver donor candidates undergoing preoperative or intraoperative liver biopsy	62/22 (72)†	Age 33 (17–61) yr<br>BMI 24.1 (17.1–31.5) kg/m^2	No NAFLD: 59<br>NAFLD: 25	13 (0–55) d
Parente, 2014, Brazil[35]	MRS-PDFF	Prospective	Patients with type 2 diabetes undergoing liver biopsy because of suspected NAFLD	13/60 (72)†	Age: 54 ± 9 yr<br>BMI: 31.4 (23.2–42.7) kg/m^2	No NAFLD: 6<br>NAFL: 40<br>NASH: 27	≤90 d
Idilman, 2013, Turkey[36]	MRI-PDFF	Retrospective	Patients undergoing liver biopsy because of suspected NAFLD	40/30	Age: 44.7 ± 13.1 yr<br>BMI: 29.9 ± 4.3 kg/m^2	No NAFLD: 7<br>NAFLD: 63	Median 14.5 (range 0–259) d

Unless otherwise specified, data are shown as means ± standard deviations, means (range), medians [25th, 75th percentiles], or as counts.
MRS-PDFF, magnetic resonance spectroscopy-proton density fat fraction; NAFL, non-alcoholic fatty liver; NAFLD, non-alcoholic fatty liver disease; NASH, non-alcoholic steatohepatitis.
* Previously unpublished data from the Helsinki MRS-PDFF cohort (see Materials and methods and Supplementary material).
† Number of participants with complete data.

FLA 5.6.0 DTD ■ JHEPR100928_proof ■ 15 November 2023 ■ 10:25 pm ■ ce

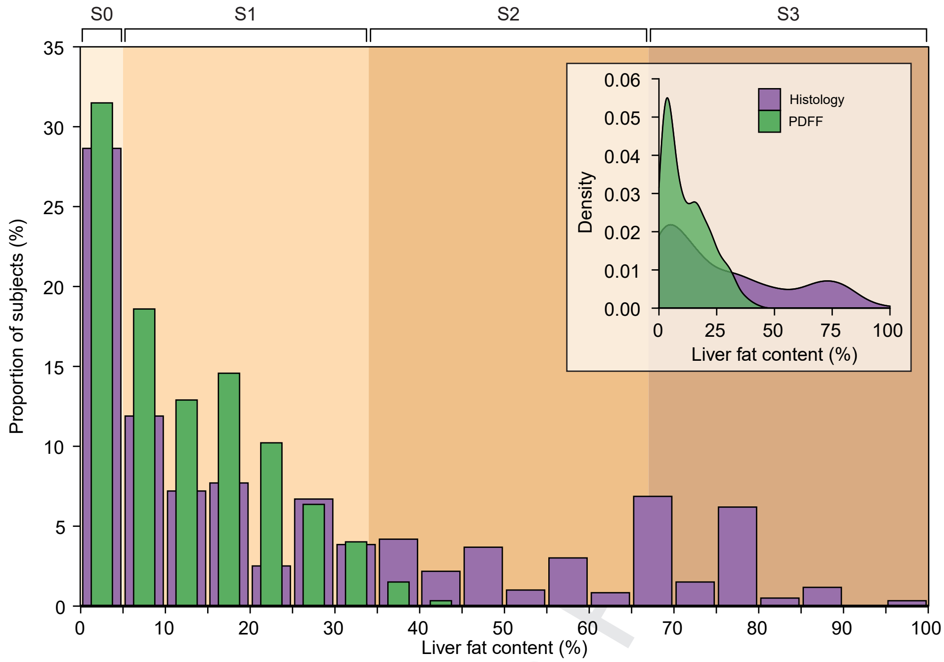

web 4C/FPO

Fig. 1. Distribution of liver fat measurements by histology and PDFF. Distribution of liver fat values in the pooled dataset of nine studies (N = 597). Dark blue bars denote histological steatosis, and the superimposed light blue bars with stripes denote PDFF. The colour-shaded background of the plot illustrates division of the x-axis into histological steatosis grades S0–S3 (S0: <5%; S1: 5–33%; S2: 34–66%; S3: >66%). The inset shows a density plot using the same data, depicting the distribution of histological steatosis and PDFF on a continuous scale (probability density function). The dark blue distribution denotes histology, and the light blue distribution denotes PDFF. PDFF, proton density fat fraction. Q9 Q10

discriminatory ability of PDFF for dichotomised histological steatosis grades (one-stage approach). Optimal rule-in thresholds were selected at the lowest value of PDFF to provide 90% specificity. For the selected thresholds, we calculated sensitivities, specificities, positive predictive values (PPV), negative predictive values (NPV), and their CIs. The AUROCs and performance parameters of the rule-in thresholds underwent 10-fold cross-validation to generate more robust, cross-validated parameters and their CIs.

Evaluation of heterogeneity and sensitivity analysis

We evaluated statistical heterogeneity using the I^2 statistic obtained from meta-analysis of Pearson correlation coefficients, in combination with Cochran's Q test. Additionally, heterogeneity was assessed in the pooled dataset using intraclass correlation coefficient, which was calculated based on the linear mixed model (see above). To evaluate different MR modalities as a potential source of between-study heterogeneity, we performed sensitivity analyses by assessing the relationship between histological steatosis and PDFF in subgroups stratified by the modality used (MRS or MRI).

Results

Study selection and risk-of-bias assessment

Fig. S1 shows the PRISMA flow diagram for study selection. We identified 3,094 potentially eligible records, which underwent screening for titles and abstracts. Out of the 293 records that finally underwent full-text screening, eight were eligible. Of these studies, two compared histology with MRI-PDFF (n = 159) and six with MRS-PDFF (n = 386). We additionally included unpublished data from 71 eligible individuals studied at our institution (the Helsinki MRS-PDFF cohort; see Materials and methods). Table 1 shows the characteristics of the nine studies included, and details regarding the MR protocols are shown in Table S4. The nine studies comprised 616 individuals (334 [54.2%] males, 282 [45.8%] females) out of which 19 had missing data (Pavlides *et al.*,[30] n = 3 because of unavailable MRS-PDFF and n = 3 as a result of unreported macrovesicular steatosis; Hwang *et al.*,[34] n = 12 and Parente *et al.*,[35] n = 1 for unknown reasons). The final dataset comprised 597 unique subjects. Q3

Most studies had a low risk of bias regarding flow and timing, index test, and patient selection (Fig. S2 and Table S5). However, reference standard risk-of-bias was deemed high for seven studies, as only Pavlides *et al.*[30] used consensus histological readings by two pathologists. Funnel plots of Pearson correlation coefficients and Bland–Altman bias estimates were symmetric and did not point to significant underlying small-study effects, with respective Egger's test *p* values of 0.28 and 0.28 (Fig. S3).

The relationship between histological steatosis and PDFF is highly linear

Fig. 1 shows the distribution of all histological and PDFF liver fat measurements in the pooled dataset. Histological steatosis ranged from 0% to 100%, whereas PDFF was distributed within a significantly narrower range and varied from 0% to 42.8%. Both distributions were positively skewed and had a numerically similar skewness and kurtosis (data not shown).

FLA 5.6.0 DTD ■ JHEPR100928_proof ■ 15 November 2023 ■ 10:25 pm ■ ce

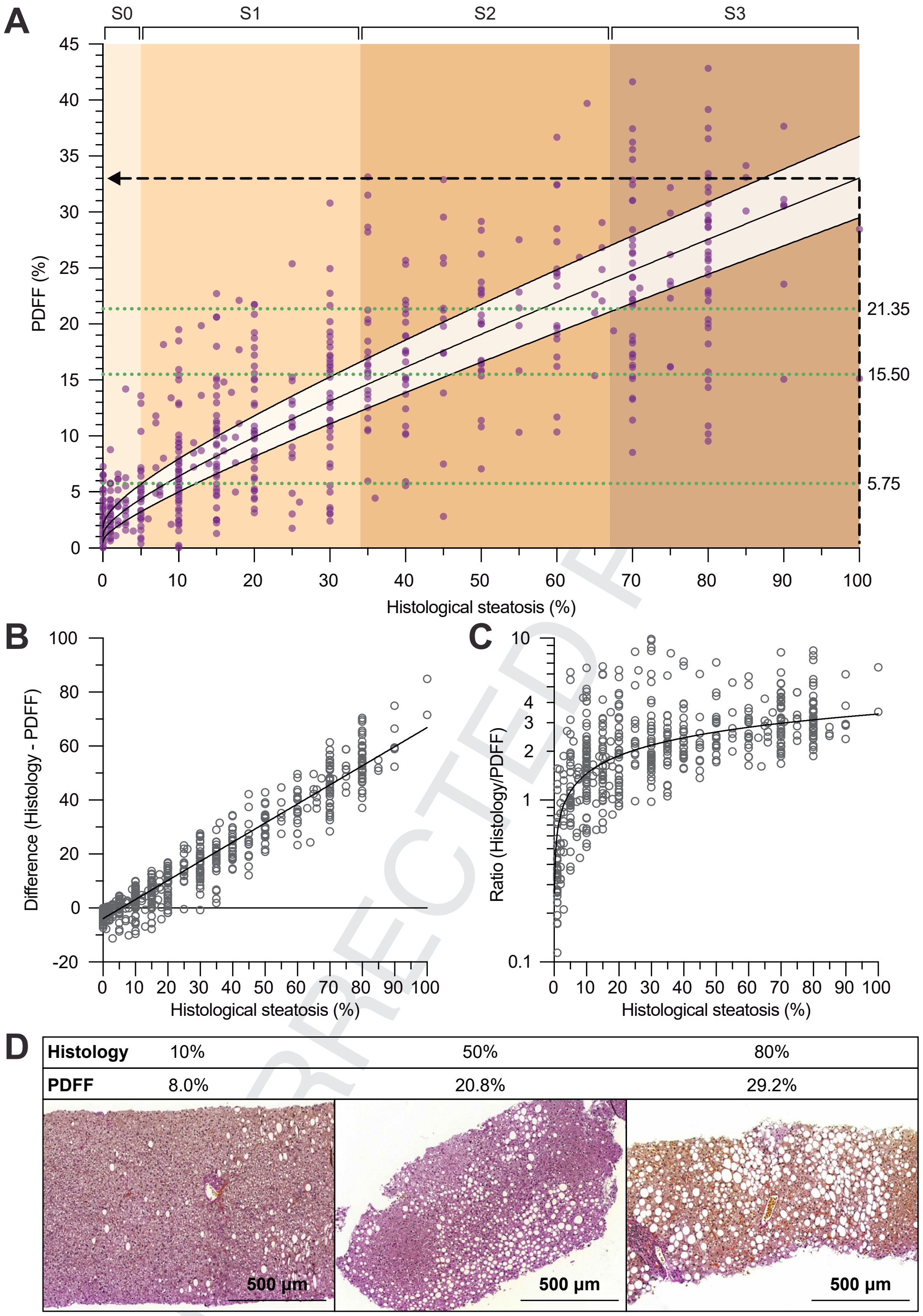

web 4C/FPO

Fig. 2. Relationship between histological steatosis fraction and PDFF. (A) Association between histological steatosis and PDFF in the pooled dataset of nine studies (N = 597). The best-fit line was determined using a linear mixed model, with study effects considered as random effects. Both variables underwent square root transformation before model fitting, and the curve fit was then back-transformed for display. The shaded area around the curve denotes 95% CI. The colour-shaded background of the plot illustrates division of the x-axis into histological steatosis grades S0–S3 (S0: <5%; S1: 5–33%; S2: 34–66%; S3: >66%). The horizontal dotted blue lines denote optimal rule-in thresholds for PDFF to predict dichotomised steatosis grades at 90% specificity (see Table 2). The dashed black lines are drawn for illustrative purposes. (B) Bland–Altman plots showing the absolute differences and (C) ratios between histological steatosis and PDFF, as a function of histological steatosis. The best-fit lines were determined using linear regression, and variables in (C) underwent logarithmic transformation before model fitting (the curve fit was then back-transformed for display). (D) Representative liver biopsies of three individuals in the Helsinki MRS-PDFF cohort. Above each image, the corresponding pathologist-reported histological steatosis fraction and PDFF are shown. Histological sections of formalin-fixed and paraffin-embedded liver biopsies underwent Herovici staining and digitisation using Pannoramic Scan 150 (3DHISTECH Ltd.; Budapest, Hungary). The images were acquired at 10 × magnification. PDFF, proton density fat fraction.

JHEP|Reports

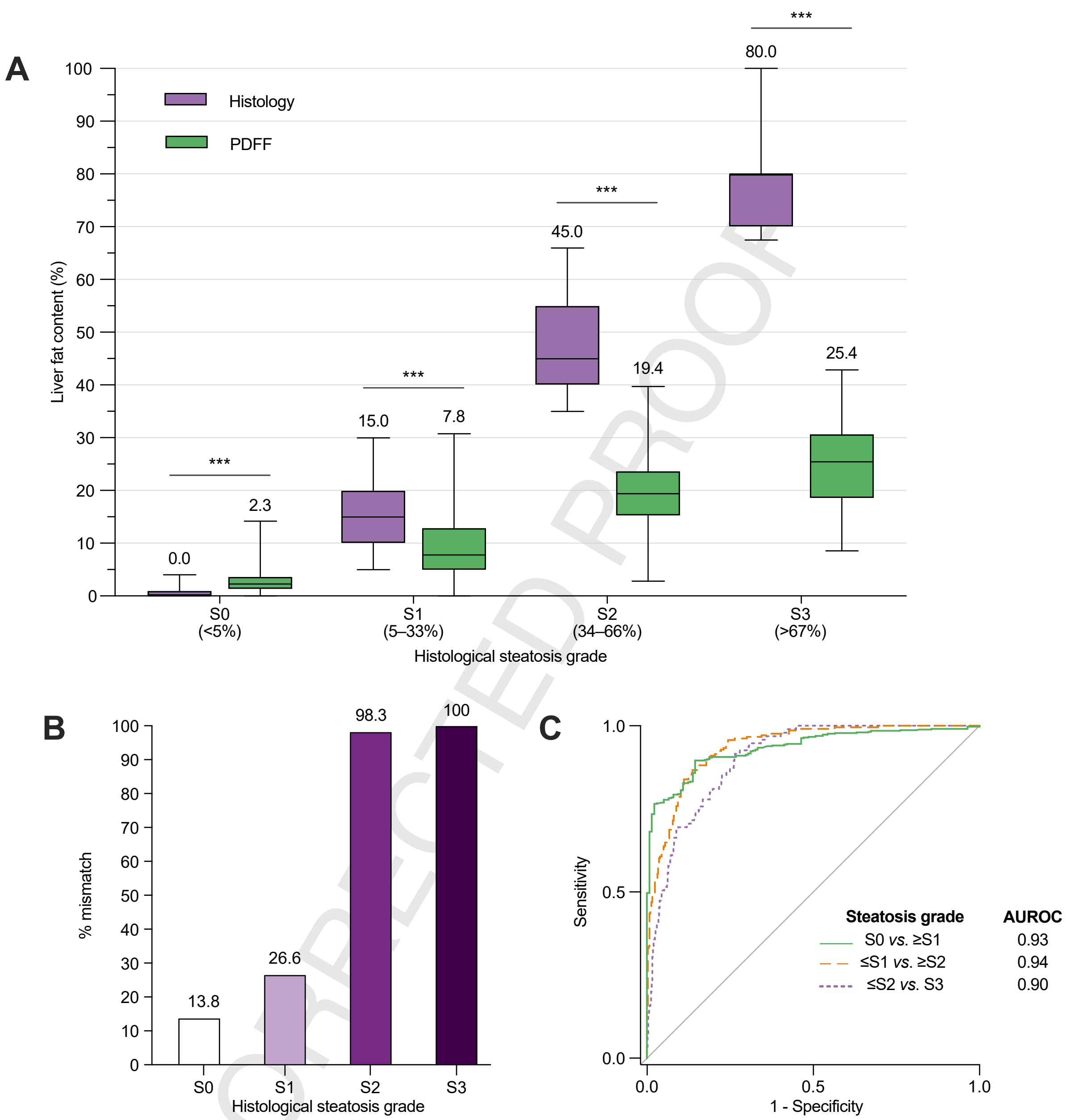

web 4C/FPO

Fig. 3. Relationship between histological steatosis grades and PDFF. (A) Distribution of histological steatosis fraction (dark blue boxes) and PDFF (light blue boxes) with respect to histological steatosis grades S0–S3. Horizontal lines within the boxes denote medians and whiskers denote minimum and maximum values. The Mann-Whitney *U* test was used. $^{***}p \leq 0.001$. (B) Proportion of individuals with steatosis grade mismatch between histology and PDFF, when PDFF is interpreted with the same grading thresholds that are conventionally used for histology (S0: <5%; S1: 5–33%; S2: 34–66%; S3: >66%). (C) Receiver operating characteristic (ROC) curves for PDFF to classify the subjects into dichotomised steatosis grades. Areas under the ROC curves (AUROC) are shown. PDFF, proton density fat fraction.

Fig. 2A shows the relationship between histological steatosis and PDFF. Except for at the lower end of liver fat content (approximately 0–10% by histology), PDFF increased highly linearly as a function of histological steatosis. The individual studies also demonstrated a considerably linear relationship, with Pearson correlation ranging from 0.72 to 0.92 (Fig. S4). Meta-analytic assessment of correlation coefficients yielded a combined estimate of 0.85 (95% CI, 0.80–0.89) (Fig. S5).

The general relationship between histological steatosis and PDFF in the pooled dataset was best described by a square root function, using the following equation (regression line in Fig. 2A):

$$\text{PDFF}\ (\%) = \left(1.0384 \pm 0.1574 + 0.4709 \pm 0.0118 \times \sqrt{\text{Histology}\ (\%)}\right)^2$$

FLA 5.6.0 DTD ■ JHEPR100928_proof ■ 15 November 2023 ■ 10:25 pm ■ ce

Corresponding values of liver fat by PDFF are markedly lower as compared with histology

At nearly every value of steatosis by histology, the corresponding PDFF was considerably lower. The histological diagnostic threshold for NAFLD at 5% represented an important inflection point below which PDFF exceeded histology and, above this point, values of PDFF were lower (Fig. 2A–C). Absolute differences between the measures increased steadily as a function of liver fat content (Fig. 2B). However, relative differences increased sharply up to approximately 10% histological steatosis and remained more constant at higher degrees of liver fat, with histological steatosis exceeding PDFF by up to 3.4-fold (Fig. 2C). On average, 100% histological steatosis corresponded to a PDFF of 33.0% (95% CI, 29.5–36.7%) (Fig. 2A). Fig. 2D shows representative histological images from three individuals with corresponding pathologist-reported and PDFF liver fat values.

Use of PDFF to classify steatosis grades requires distinct thresholds

Steatosis grades S0–S3 are frequently used to quantify histological liver fat (S0: <5%; S1: 5–33%; S2: 34–66%; S3: >66%). Consistent with our findings above, PDFF was significantly higher compared with histological steatosis fraction in individuals with grade S0, while being significantly lower in subjects with grades S1 to S3 (Fig. 3A). Median PDFF values in individuals with histological steatosis grades S0, S1, S2, and S3 were 2.3%, 7.8%, 19.4%, and 25.4%, respectively (Fig. 3A). In accordance, use of PDFF to predict steatosis grades with the thresholds that are commonly used for histology led to a gross mismatch between the actual and predicted steatosis grades, especially for individuals with grades S2–S3 (Fig. 3B and Table S6).

Despite the significant disagreement between histological steatosis and PDFF in terms of absolute values, ROC analysis revealed a remarkably high discriminatory ability for PDFF to classify dichotomised steatosis grades (Fig. 3C). Cross-validated AUROCs (± standard errors) were 0.94 ± 0.02 for S0 *vs.* S1–S3, 0.94 ± 0.03 for S0–S1 *vs.* S2–S3, and 0.91 ± 0.04 for S0–S2 *vs.* S3. Targeting at a specificity of 90%, optimal PDFF rule-in thresholds to classify steatosis grades were ≥5.75% for S1 or higher (*i.e.* a diagnosis of NAFLD), ≥15.50% for S2 or higher (moderate-to-severe steatosis), and ≥21.35% for S3 (severe steatosis). Table 2 shows cross-validated diagnostic performance parameters for these thresholds in the pooled dataset. Raw performance parameters, and additional rule-in and rule-out thresholds for 90/95% sensitivity/specificity, are shown in Table S7.

Between-study heterogeneity and sensitivity analysis

The included studies demonstrated a moderate-to-substantial degree of heterogeneity with respect to observed linearity between histological steatosis and PDFF (I^2 = 67.0% [95% CI, 33.3–83.7%], p <0.01; Fig. S5). In the linear mixed model of pooled data (Fig. 2A), the proportion of variance attributable to between-study differences in the relationship between histological steatosis and PDFF was 28.9% (intraclass correlation coefficient). Regression lines fit to individual study data showed variable slopes, but this variability was random across the different MR modalities (MRS or MRI) (Fig. S6). In a sensitivity analysis, the data for MRS-PDFF and MRI-PDFF showed a complete overlap, with best-fit lines having a near-identical association with histological steatosis (Fig. S7). Thus, heterogeneity likely originated from interrater variability related to histological steatosis assessment.

The non-hepatocyte cell fraction as a potential confounder of liver fat measurement

To determine whether a significant non-hepatocyte cell fraction may act as a confounder with respect to liver fat measurement by histology *vs.* PDFF, we determined the size of this fraction in liver biopsies from 138 individuals. The RNA-seq-based analysis of hepatic cell-type composition identified six distinct cell populations. The average proportion of hepatocytes was 58.5 ± 5.2% (Fig. 4A), and the fraction of hepatocytes had a significantly negative correlation with liver fat content (r_s = -0.21, p <0.05) (Fig. 4B). This finding provides one explanation as to why PDFF values are lower compared with histopathology, as the latter only considers hepatocytes in deriving the steatosis fraction.

Discussion

We pooled patient-level measurements of liver fat assessed by histology and PDFF from 597 individuals across nine studies. Our principal finding was that, as a function of steatosis, both absolute and relative differences between the two methods increased markedly. Compared with histological steatosis fraction, values of PDFF for the same individuals were significantly lower (Fig. 2A–D). The highest recorded value of histological steatosis was 100%, whereas the highest PDFF was only 42.8%. This was despite the methods having considerable (albeit non-perfect) linearity and seemingly measuring liver fat content in the same units, that is, percentages.

PDFF slightly exceeded histological steatosis in the lowest range of liver fat below 5% (Fig. 3A). In the normal human liver without histologically visible lipid droplets, biochemically measured triacylglycerols constitute 2–6% of wet tissue weight.[37–39] This amount of lipid is quantifiable by PDFF but would be invisible to the pathologist. Thus, as we observed, PDFF would predictably be higher in the <5% range. At 5% liver fat, which fortuitously is the histological diagnostic threshold for NAFLD, histology and PDFF approximately coincided. Above the inflection point of 5%, however, histological steatosis was consistently and up to over threefold higher. On average, 100% steatosis by histology corresponded to a PDFF of 33%.

Fig. 5 illustrates how the principles underlying liver fat assessment by histology and PDFF are fundamentally different. The pathologist visualises a histological cross-section and

Table 2. Thresholds and 10-fold cross-validated diagnostic performance parameters for PDFF to predict dichotomised histological steatosis grades at 90% specificity in the pooled cohort.

Steatosis grade classification	Threshold	Se, %(95% CI)	Sp, % (95% CI)	PPV, (95% CI)	NPV, % (95% CI)
S0 *vs.* S1–S3	≥5.75	79.5 (77.2–81.8)	90.1 (85.7–96.1)	96.6 (94.8–98.4)	57.1 (52.9–61.2)
S0–S1 *vs.* S2–S3	≥15.50	78.8 (73.6–83.9)	90.1 (87.2–93.1)	81.7 (77.0–86.4)	88.6 (85.7–91.5)
S0–S2 *vs.* S3	≥21.35	69.0 (59.9–78.1)	90.0 (88.6–91.5)	56.7 (52.8–60.5)	94.0 (92.5–95.6)

NPV, negative predictive value; PDFF, proton density fat fraction; PPV, positive predictive value; Se, sensitivity; Sp, specificity.

UNCORRECTED PROOF

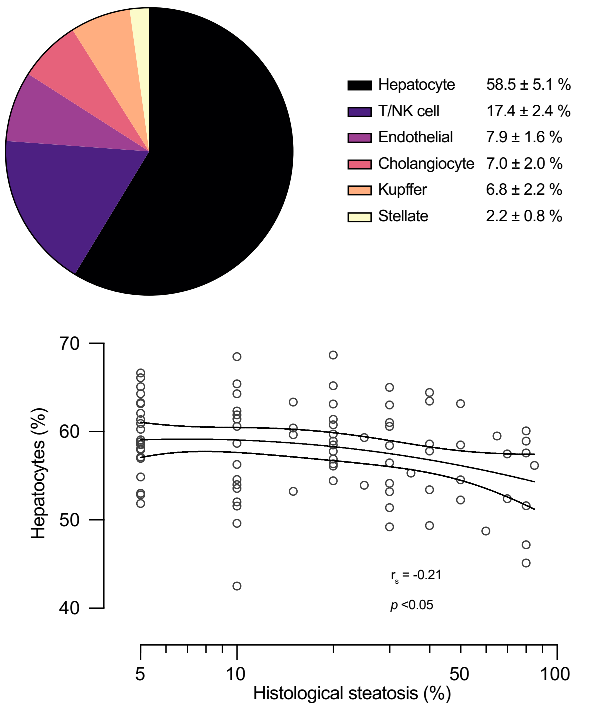

web 4C/FPO

Fig. 4. The human liver cell-type composition. (A) Average proportions of the major hepatic cell-type fractions, as determined by the RNA-seq-based CIBERSORTx analysis in 138 human liver biopsies. Data are shown as mean ± SD. (B) Association between histological steatosis and the fraction of hepatocytes in human liver biopsies. Regression line was fit using a quadratic model after log-transforming the liver fat fraction. The dashed lines denote 95% CI. The Spearman correlation coefficient is shown. NK, natural killer.

estimates the proportion of macrovesicular lipid droplet-containing hepatocytes out of all hepatocytes, which can range from 0% to 100%. This scale is inherently semi-quantitative and disregards changes in size of the lipid droplets. In contrast, PDFF quantifies fat within a sampled liver volume, based on the measured density of mobile protons in fatty acids out of the total mobile proton densities of fatty acids and water (Fig. 5). Protons originating from membrane lipid-incorporated fatty acids are opaque to MR, and thus the MR-visible fat-attributable protons mainly represent triacylglycerols.[40] Because the denominator in PDFF includes tissue water residing in all cells and within the extracellular space—and because excess triacylglycerol only accumulates inside of hepatocytes—liver PDFF should never reach 100%. The highest PDFF of 42.8% in the present analysis is similar to the maximum of 47.5% reported in the Dallas Heart Study with 2,287 individuals.[9] Even in the most severe cases of fatty liver in which most or all hepatocytes contain macrovesicular lipid droplets in histology, biochemically measured lipid content rarely exceeds 40%.[37]

In addition to hepatocytes, the hepatic volume fraction probed by MRS and MRI contains a variety of other cell types, which also contain water and presumably affect PDFF by contributing to the denominator. Using a state-of-the-art RNA-seq method to estimate the human liver cell-type composition, we found that hepatocytes comprised less than 60% of all cells on average (Fig. 4A). Although this analysis discounts volume differences between cells (hepatocytes are among the largest hepatic cells) and extracellular water was not measured, the high proportion of non-parenchymal cells may partly explain the discrepancy between histological steatosis and PDFF. Interestingly, and despite the low prevalence of advanced liver fibrosis in the RNA-seq cohort, higher liver fat was associated with a slight but significant decrease in the proportion of hepatocytes (Fig. 4B). This finding is novel and may point to an early degradation of hepatocyte viability already in the initial stages of NAFLD.

Owing to poor agreement between the absolute values of PDFF and histological steatosis, the standard thresholds to classify steatosis grades were unapplicable for PDFF (Fig. 3B). We successfully derived optimal thresholds for PDFF to classify dichotomised histological steatosis grades (Table 2 and Table S7). The PDFF rule-in threshold to predict steatosis grade ≥S1 (*i.e.* histological steatosis ≥5%, or NAFLD) at a specificity of 90.1% and PPV of 96.6% was ≥5.75%. This finding is in line with the currently widely adopted PDFF definition of ≥5.56% for NAFLD, which was derived in the population-based Dallas Heart Study without liver histology information, based on the 95th percentile PDFF in normal-weight individuals without a history of liver disease or metabolic risk factors.[9] Comparable albeit variable PDFF thresholds have been found previously in small NAFLD liver biopsy cohorts.[14–16,29,33] Our large multi-centre analysis is the first to provide robust and likely well-generalisable estimates. It is, however, challenging to accurately define the upper limit of normal for PDFF. Use of pathologists' interpretation as the reference standard is problematic, as inter-rater variability likely introduces some bias in all estimates.[24] The relationship between histology and PDFF was also less linear in the 0–10% range (Fig. 2A). An alternative approach could be to determine a level of PDFF associated with a clinically significant increase in adverse liver-related outcomes.

The main limitation of this study relates to methodological variability in liver fat assessment. Compared with histology, PDFF represents an inherent physical tissue property, is observer-independent, and is measured within a much larger volume **Q13** compartment. It does, however, lack standardisation, as is evident from variability in the reported MR protocol-related parameters (Table S4). We carefully examined the MR protocols of each study to ascertain that the most important sources of bias were likely accounted for.[7] In a sensitivity analysis, MRS-PDFF and MRI-PDFF showed strikingly concordant results (Fig. S7), which is in keeping with the meta-analysis by Yokoo *et al.*[8] Moreover, PDFF has been found to be consistent across different imaging centres, scanner manufacturers, field strengths, and reconstruction methods.[41] Individual-related factors such as age, sex, or BMI do not significantly influence PDFF quantification.[42]

Histological assessment of steatosis is subjective and inherently semiquantitative, bearing several well-known limitations such as inter-rater error and the biopsy-associated sampling error.[43,44] Across the included studies, liver histology was analysed by nine different pathologists. This likely introduced the greatest degree of bias in our analysis by manifesting as between-study heterogeneity. In the pooled data, PDFF exhibited moderate variance at each degree of histological steatosis (Fig. 2A), which was less pronounced at the individual-study level (Fig. S4). Despite of this variability, differences between the two methods consistently increased as a function of liver fat in both absolute (Fig. 2B) and relative (Fig. 2C) terms. This phenomenon was readily observable in all individual study data (Fig. S4). In recent years, digital image analysis of histology has gained popularity in

UNCORRECTED PROOF

FLA 5.6.0 DTD ■ JHEPR100928_proof ■ 15 November 2023 ■ 10:25 pm ■ ce

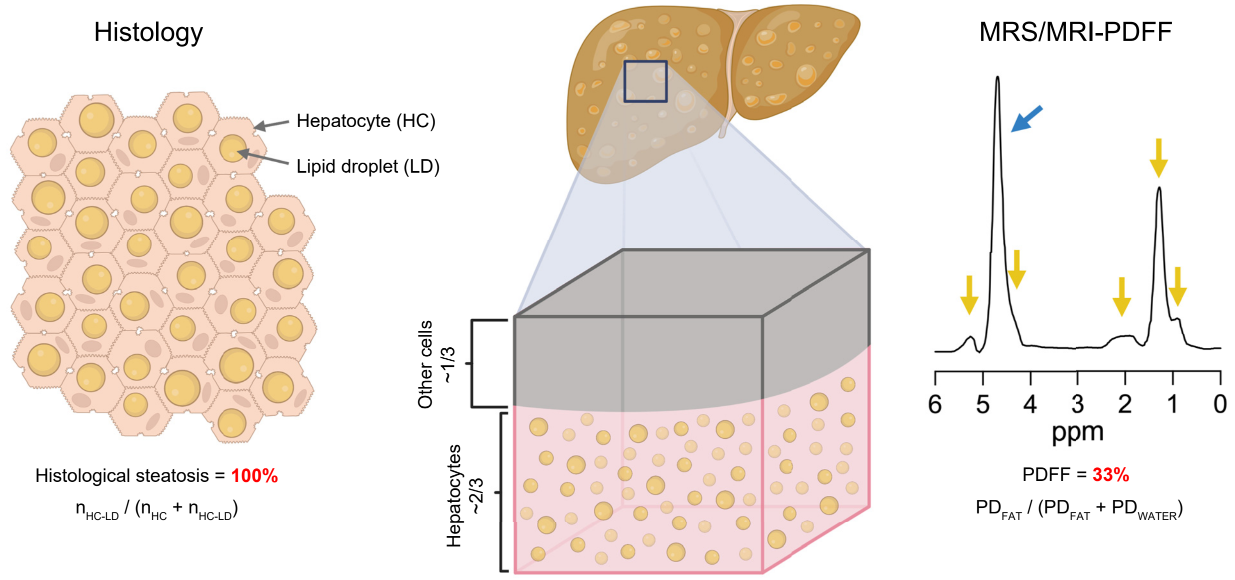

web 4C/FPO

Fig. 5. Simplified illustration of key differences in liver fat measurement between histology and PDFF. In fatty liver disease, lipid droplets accumulate in the cytoplasm of hepatocytes, which represent the principal tissue cell type. However, cells other than hepatocytes comprise approximately one-third of the total hepatic cell population. In histological analysis of steatosis, a pathologist visually estimates liver fat as the fraction of macrovesicular lipid droplet-containing hepatocytes ($n_{HC\text{-}LD}$), out of all visible hepatocytes ($n_{HC} + n_{HC\text{-}LD}$) within a histological cross-section. A 100% steatosis is reached when all the visible hepatocytes contain macrovesicular lipid droplets (exemplified on the left). However, liver fat as measured by MRS/MRI-PDFF is calculated by dividing the MR-visible proton density of tissue fat (PD_{FAT}) by the sum of proton densities of tissue fat and water ($PD_{FAT} + PD_{WATER}$). PDFF takes into account the spectral complexity of fat, including the smaller fat peaks (arrows in yellow) relative to that of water (arrow in blue). In the present study, 100% histological steatosis corresponded to an average PDFF of 33%. A representative MR spectrum from a patient with a PDFF of 33% is shown on the right. In addition to hepatocytes, the tissue volume fraction probed by MRS/MRI contains other cell types as well as components of the extracellular matrix, influencing the MR-visible water-to-fat ratio and presumably contributing to the numerical difference between PDFF and histological steatosis. MR, magnetic resonance; MRI-PDFF, magnetic resonance imaging-proton density fat fraction; MRS, magnetic resonance spectroscopy.

UNCORRECTED

quantifying steatosis, especially in clinical trials.[45] These methods usually quantify steatosis as the percentage image area occupied by lipid droplets and are thereby expected to deviate from the semi-quantitative assessment by pathologists. Because computerised analysis eliminates human variability, it would likely render the relationship of histological steatosis and PDFF more comparable across different centres. Future studies should investigate whether this is the case and determine the linearity and agreement between PDFF and image analysis-acquired histological steatosis fraction.

Given that histological steatosis and PDFF share a similar diagnostic threshold for NAFLD, what, then, are the clinical implications of our findings? In longitudinal studies with registry-based outcome data, the only baseline feature of NAFLD consistently predicting liver-related mortality is fibrosis.[46] However, paired-biopsy studies have shown that the higher the degree of liver fat is at baseline, the more likely is fibrosis onset or progression during follow-up.[47–49] However, a ≥30% decrease in PDFF predicts fibrosis regression, which may be a useful marker in cases where liver biopsy is not clinically indicated and non-invasive measures of fibrosis, such as MR elastography, are unavailable.[50] Therefore, steatosis, while perhaps not prognostic by itself, is a relevant predictor of disease progression and regression. We found that disregarding the differences between PDFF and histology would lead to a gross misclassification of especially those patients with severe steatosis. The future clinician is likely to be confronted with information from different types of exams, as liver biopsy and PDFF may be used in parallel or sequentially during diagnosis and follow-up. This adds a layer of complexity in clinical decision-making. For example, if PDFF is used to assess treatment effect after an initial liver biopsy, lack of consideration of methodological differences may lead to an illusion of significant improvement in liver fat. However, if biopsy and imaging were performed in parallel, their results could appear conflicting. Future guidelines for NAFLD should emphasise that histology and PDFF represent fundamentally different methods of liver fat quantification, while underlining that the former may yield values in excess of three times higher. This is important considering the near-term increase in the use of MRI-PDFF in particular, in routine patient care. Q4

Abbreviations

AUROC, area under the receiver operating characteristic curve; CENTRAL, Cochrane central register of controlled trials; HC, hepatocyte; LD, lipid droplet; MEDLINE, medical literature analysis and retrieval system online; MR, magnetic resonance; MRI, magnetic resonance imaging; MRS, magnetic resonance spectroscopy; NAFL, non-alcoholic fatty liver; NAFLD,

non-alcoholic fatty liver disease; NASH, non-alcoholic steatohepatitis; NPV, negative predictive value; PD, proton density; PDFF, proton density fat fraction; PPV, positive predictive value; PRISMA, Preferred Reporting Items for Systematic Reviews and Meta-analyses; QUADAS, Quality Assessment of Diagnostic Accuracy Studies; ROC, receiver operating characteristic.

Financial support

HYJ was supported by grants from the Academy of Finland (no. 309263), the Novo Nordisk Foundation, and the Sigrid Jusélius Foundation. SQ was supported by grants from the Orion Research Foundation, the Yrjö Jahnsson Foundation, the Maud Kuistila Memorial Foundation, the Emil Aaltonen Foundation, and the Finnish Medical Foundation. TT was supported by grants from the Academy of Finland (no. 315589 and no. 320129) and the Sigrid Jusélius Foundation. RL receives funding support from NCATS (5UL1TR001442), NIDDK (U01DK061734, U01DK130190, R01DK106419, R01DK121378, R01DK124318, P30DK120515), NHLBI Q5 (P01HL147835), and NIAAA (U01AA029019). AT was supported by a Senior Clinical Research Scholarship from the Fonds de recherche du Québec en Santé and Fondation de l'association des radiologistes du Québec Clinical Research Scholarship (FRQS-ARQ #298509). MP acknowledges support from the Oxford NIHR Biomedical Research Centre.

Conflict of interest

RL serves as a consultant to Aardvark Therapeutics, Altimmune, Anylam/Regeneron, Amgen, Arrowhead Pharmaceuticals, AstraZeneca, Bristol-Myers Squibb, CohBar, Eli Lilly, Galmed, Gilead, Glympse bio, Hightide, Inipharm, Intercept, Inventiva, Ionis, Janssen Inc., Madrigal, Metacrine Inc., NGM Biopharmaceuticals, Novartis, Novo Nordisk, Merck, Pfizer, Sagimet, Theratechnologies, 89 bio, Terns Pharmaceuticals, and Viking Therapeutics. In addition, RL's institutions received research grants from Arrowhead Pharmaceuticals, AstraZeneca, Boehringer-Ingelheim, Bristol-Myers Squibb, Eli Lilly, Galectin Therapeutics, Galmed Pharmaceuticals, Gilead, Hanmi, Intercept, Inventiva, Ionis, Janssen, Madrigal Pharmaceuticals, Merck, NGM Biopharmaceuticals, Novo Nordisk, Pfizer, Sonic Incytes, and Terns Pharmaceuticals. RL is a co-founder of LipoNexus Inc. CBS reports research grants from ACR, Bayer, GE, Gilead, Pfizer, Philips, Siemens; equipment loans from GE; lab service agreements with Enanta, Gilead, ICON, Intercept, Nusirt, Shire, Synageva, Takeda; institutional consulting for BMS, Exact Sciences, IBM-Watson, Pfizer; Personal consulting for Blade, Boehringer, Epigenomics, and Guerbet; receipt of royalties and/or honoraria from Medscape and Wolters Kluwer; ownership of stock options in Livivos; unpaid advisory board position in Quantix Bio. CBS serves as Chief Medical Officer for Livivos (unsalaried position). Unrelated to this paper, MP declares stock ownership in Perspectum Ltd. All other authors state that no conflicts of interest regarding this manuscript exist.

Please refer to the accompanying ICMJE disclosure forms for further details.

Authors' contributions

Designed the study, performed the literature review, and screened records for inclusion: SQ, HYJ. Acquired data for the meta-analysis, interpreted the data, and wrote the manuscript: SQ. Performed statistical analysis: SQ, ML. Performed RNA-seq analyses: EV, TT. Performed clinical studies: KP, PA. Performed liver biopsies: AJ, AKP. Analysed liver histology of the Helsinki MRS-PDFF cohort and the liver RNA-seq cohort: JA. Analysed and interpreted magnetic resonance data of the Helsinki MRS-PDFF cohort: JD, TEL, WS. Provided data for the United States cohort: AT, RL, CBS. Provided data for the United Kingdom cohort: MP. Provided data for the Turkey cohort: ISI, MK. Provided data for the Netherlands cohort: JHR, JS. Supervised the study: HYJ. Critically revised the manuscript draft for important intellectual content and approved the final manuscript: all Q6 authors.

Data availability statement

The datasets generated and/or analysed during the current study are not publicly available but are available from the corresponding authors on reasonable request.

Acknowledgements

We gratefully acknowledge the volunteers for their help and thank Aila Karioja-Kallio and Päivi Ihamuotila for their excellent technical assistance. Fig. 5 was created with BioRender.com. Open access funded by Helsinki University Library.

Supplementary data

Supplementary data to this article can be found online at https://doi.org/10.1016/j.jhepr.2023.100928.

References Q7Q8

Author names in bold designate shared co-first authorship

[1] Tiniakos DG, Vos MB, Brunt EM. Nonalcoholic fatty liver disease: pathology and pathogenesis. Annu Rev Pathol 2010;5:145–171.

[2] Chalasani N, Younossi Z, Lavine JE, Charlton M, Cusi K, Rinella M, et al. The diagnosis and management of nonalcoholic fatty liver disease: practice guidance from the American Association for the Study of Liver Diseases. Hepatology 2018;67:328–357.

[3] European Association for the Study of the Liver, European Association for the Study of Diabetes, European Association for the Study of Obesity. EASL-EASD-EASO Clinical Practice Guidelines for the management of non-alcoholic fatty liver disease. J Hepatol 2016;64:1388–1402.

[4] Eslam M, Sarin SK, Wong VW, Fan JG, Kawaguchi T, Ahn SH, et al. The Asian Pacific Association for the Study of the Liver clinical practice guidelines for the diagnosis and management of metabolic associated fatty liver disease. Hepatol Int 2020;14:889–919.

[5] Burt AD, Portmann BC, Ferrell LD. MacSween's Pathology of the liver. 5 edn. London: Churchill Livingstone/Elsevier; 2007.

[6] Caussy C, Reeder SB, Sirlin CB, Loomba R. Noninvasive, quantitative assessment of liver fat by MRI-PDFF as an endpoint in NASH trials. Hepatology 2018;68:763–772.

[7] Reeder SB, Cruite I, Hamilton G, Sirlin CB. Quantitative assessment of liver fat with magnetic resonance imaging and spectroscopy. J Magn Reson Imaging 2011;34:729–749.

[8] Yokoo T, Serai SD, Pirasteh A, Bashir MR, Hamilton G, Hernando D, et al. Linearity, bias, and precision of hepatic proton density fat fraction measurements by using MR imaging: a meta-analysis. Radiology 2018;286:486–498.

[9] Szczepaniak LS, Nurenberg P, Leonard D, Browning JD, Reingold JS, Grundy S, et al. Magnetic resonance spectroscopy to measure hepatic triglyceride content: prevalence of hepatic steatosis in the general population. Am J Physiol Endocrinol Metab 2005;288:E462–E468.

[10] Noureddin M, Lam J, Peterson MR, Middleton M, Hamilton G, Le TA, et al. Utility of magnetic resonance imaging versus histology for quantifying changes in liver fat in nonalcoholic fatty liver disease trials. Hepatology 2013;58:1930–1940.

[11] Loomba R, Neuschwander-Tetri BA, Sanyal A, Chalasani N, Diehl AM, Terrault N, et al. Multicenter validation of association between decline in MRI-PDFF and histologic response in NASH. Hepatology 2020;72:1219–1229.

[12] McPherson S, Jonsson JR, Cowin GJ, O'Rourke P, Clouston AD, Volp A, et al. Magnetic resonance imaging and spectroscopy accurately estimate the severity of steatosis provided the stage of fibrosis is considered. J Hepatol 2009;51:389–397.

[13] Permutt Z, Le TA, Peterson MR, Seki E, Brenner DA, Sirlin C, Loomba R. Correlation between liver histology and novel magnetic resonance imaging in adult patients with non-alcoholic fatty liver disease – MRI accurately quantifies hepatic steatosis in NAFLD. Aliment Pharmacol Ther 2012;36:22–29.

[14] Park CC, Nguyen P, Hernandez C, Bettencourt R, Ramirez K, Fortney L, et al. Magnetic resonance elastography vs transient elastography in detection of fibrosis and noninvasive measurement of steatosis in patients with biopsy-proven nonalcoholic fatty liver disease. Gastroenterology 2017;152:598–607.e592.

[15] Middleton MS, Heba ER, Hooker CA, Bashir MR, Fowler KJ, Sandrasegaran K, et al. Agreement between magnetic resonance imaging proton density fat fraction measurements and pathologist-assigned steatosis grades of liver biopsies from adults with nonalcoholic steatohepatitis. Gastroenterology 2017;153:753–761.

[16] Tang A, Tan J, Sun M, Hamilton G, Bydder M, Wolfson T, et al. Nonalcoholic fatty liver disease: MR imaging of liver proton density fat fraction to assess hepatic steatosis. Radiology 2013;267:422–431.

UNCORRECTED PROOF

[17] Yokoo T, Shiehmorteza M, Hamilton G, Wolfson T, Schroeder ME, Middleton MS, et al. Estimation of hepatic proton-density fat fraction by using MR imaging at 3.0 T. Radiology 2011;258:749–759.
[18] Reeder SB, Sirlin CB. Quantification of liver fat with magnetic resonance imaging. Magn Reson Imaging Clin N Am 2010;18:337–357.
[19] Yokoo T, Bydder M, Hamilton G, Middleton MS, Gamst AC, Wolfson T, et al. Nonalcoholic fatty liver disease: diagnostic and fat-grading accuracy of low-flip-angle multiecho gradient-recalled-echo MR imaging at 1.5 T. Radiology 2009;251:67–76.
[20] European Association for the Study of the Liver. EASL Clinical Practice Guidelines on non-invasive tests for evaluation of liver disease severity and prognosis – 2021 update. J Hepatol 2021;75:659–689.
[21] Page MJ, McKenzie JE, Bossuyt PM, Boutron I, Hoffmann TC, Mulrow CD, et al. The PRISMA 2020 statement: an updated guideline for reporting systematic reviews. BMJ 2021;372:n71.
[22] Ouzzani M, Hammady H, Fedorowicz Z, Elmagarmid A. Rayyan-a web and mobile app for systematic reviews. Syst Rev 2016;5:210.
[23] Whiting PF, Rutjes AW, Westwood ME, Mallett S, Deeks JJ, Reitsma JB, et al. QUADAS-2: a revised tool for the quality assessment of diagnostic accuracy studies. Ann Intern Med 2011;155:529–536.
[24] **El-Badry AM**, **Breitenstein S**, Jochum W, Washington K, Paradis V, Rubbia-Brandt L, et al. Assessment of hepatic steatosis by expert pathologists: the end of a gold standard. Ann Surg 2009;250:691–697.
[25] Newman AM, Steen CB, Liu CL, Gentles AJ, Chaudhuri AA, Scherer F, et al. Determining cell type abundance and expression from bulk tissues with digital cytometry. Nat Biotechnol 2019;37:773–782.
[26] **MacParland SA**, **Liu JC**, **Ma XZ**, Innes BT, Bartczak AM, Gage BK, et al. Single cell RNA sequencing of human liver reveals distinct intrahepatic macrophage populations. Nat Commun 2018;9:4383.
[27] Balduzzi S, Rucker G, Schwarzer G. How to perform a meta-analysis with R: a practical tutorial. Evid Based Ment Health 2019;22:153–160.
[28] Bates D, Machler M, Bolker BM, Walker SC. Fitting linear mixed-effects models using lme4. J Stat Softw 2015;67:1–48.
[29] Runge JH, Smits LP, Verheij J, Depla A, Kuiken SD, Baak BC, et al. MR spectroscopy-derived proton density fat fraction is superior to controlled attenuation parameter for detecting and grading hepatic steatosis. Radiology 2018;286:547–556.
[30] Pavlides M, Banerjee R, Tunnicliffe EM, Kelly C, Collier J, Wang LM, et al. Multiparametric magnetic resonance imaging for the assessment of non-alcoholic fatty liver disease severity. Liver Int 2017;37:1065–1073.
[31] Traussnigg S, Kienbacher C, Gajdosik M, Valkovic L, Halilbasic E, Stift J, et al. Ultra-high-field magnetic resonance spectroscopy in non-alcoholic fatty liver disease: novel mechanistic and diagnostic insights of energy metabolism in non-alcoholic steatohepatitis and advanced fibrosis. Liver Int 2017;37:1544–1553.
[32] Rastogi R, Gupta S, Garg B, Vohra S, Wadhawan M, Rastogi H. Comparative accuracy of CT, dual-echo MRI and MR spectroscopy for preoperative liver fat quantification in living related liver donors. Indian J Radiol Imaging 2016;26:5–14.
[33] Tang A, Desai A, Hamilton G, Wolfson T, Gamst A, Lam J, et al. Accuracy of MR imaging-estimated proton density fat fraction for classification of dichotomized histologic steatosis grades in nonalcoholic fatty liver disease. Radiology 2015;274:416–425.
[34] Hwang I, Lee JM, Lee KB, Yoon JH, Kiefer B, Han JK, Choi BI. Hepatic steatosis in living liver donor candidates: preoperative assessment by using breath-hold triple-echo MR imaging and 1H MR spectroscopy. Radiology 2014;271:730–738.
[35] Parente DB, Rodrigues RS, Paiva FF, Oliveira Neto JA, Machado-Silva L, Lanzoni V, et al. Is MR spectroscopy really the best MR-based method for the evaluation of fatty liver in diabetic patients in clinical practice? PLoS One 2014;9:e112574.
[36] Idilman IS, Aniktar H, Idilman R, Kabacam G, Savas B, Elhan A, et al. Hepatic steatosis: quantification by proton density fat fraction with MR imaging versus liver biopsy. Radiology 2013;267:767–775.
[37] Gillman J, Gillman T. Perspectives in human malnutrition: a contribution to the biology of disease from a clinical and pathological study of chronic malnutrition and pellagra in the african. New York: Grune & Stratton; 1951.
[38] Roldan-Valadez E, Favila R, Martinez-Lopez M, Uribe M, Rios C, Mendez-Sanchez N. In vivo 3T spectroscopic quantification of liver fat content in nonalcoholic fatty liver disease: correlation with biochemical method and morphometry. J Hepatol 2010;53:732–737.
[39] Li M, Song J, Mirkov S, Xiao SY, Hart J, Liu W. Comparing morphometric, biochemical, and visual measurements of macrovesicular steatosis of liver. Hum Pathol 2011;42:356–360.
[40] Hakumaki JM, Kauppinen RA. 1H NMR visible lipids in the life and death of cells. Trends Biochem Sci 2000;25:357–362.
[41] **Hu HH**, **Yokoo T**, Bashir MR, Sirlin CB, Hernando D, Malyarenko D, et al. Linearity and bias of proton density fat fraction as a quantitative imaging biomarker: a multicenter, multiplatform, multivendor phantom study. Radiology 2021;298:640–651.
[42] Heba ER, Desai A, Zand KA, Hamilton G, Wolfson T, Schlein AN, et al. Accuracy and the effect of possible subject-based confounders of magnitude-based MRI for estimating hepatic proton density fat fraction in adults, using MR spectroscopy as reference. J Magn Reson Imaging 2016;43:398–406.
[43] Younossi ZM, Gramlich T, Liu YC, Matteoni C, Petrelli M, Goldblum J, et al. Nonalcoholic fatty liver disease: assessment of variability in pathologic interpretations. Mod Pathol 1998;11:560–565.
[44] **Ratziu V**, **Charlotte F**, Heurtier A, Gombert S, Giral P, Bruckert E, et al. Sampling variability of liver biopsy in nonalcoholic fatty liver disease. Gastroenterology 2005;128:1898–1906.
[45] Dinani AM, Kowdley KV, Noureddin M. Application of artificial intelligence for diagnosis and risk stratification in NAFLD and NASH: the state of the art. Hepatology 2021;74:2233–2240.
[46] **Ekstedt M**, **Hagström H**, Nasr P, Fredrikson M, Stål P, Kechagias S, Hultcrantz R. Fibrosis stage is the strongest predictor for disease-specific mortality in NAFLD after up to 33 years of follow-up. Hepatology 2015;61:1547–1554.
[47] Ekstedt M, Franzen LE, Mathiesen UL, Thorelius L, Holmqvist M, Bodemar G, Kechagias S. Long-term follow-up of patients with NAFLD and elevated liver enzymes. Hepatology 2006;44:865–873.
[48] Pais R, Charlotte F, Fedchuk L, Bedossa P, Lebray P, Poynard T, et al. A systematic review of follow-up biopsies reveals disease progression in patients with non-alcoholic fatty liver. J Hepatol 2013;59:550–556.
[49] Ajmera V, Park CC, Caussy C, Singh S, Hernandez C, Bettencourt R, et al. Magnetic resonance imaging proton density fat fraction associates with progression of fibrosis in patients with nonalcoholic fatty liver disease. Gastroenterology 2018;155:307–310.e302.
[50] Tamaki N, Munaganuru N, Jung J, Yonan AQ, Loomba RR, Bettencourt R, et al. Clinical utility of 30% relative decline in MRI-PDFF in predicting fibrosis regression in non-alcoholic fatty liver disease. Gut 2022;71:983–990.

UNCORRECTED PROOF

FLA 5.6.0 DTD ■ JHEPR100928_proof ■ 15 November 2023 ■ 10:25 pm ■ ce

Journal of Hepatology, Volume ■

Supplemental information

Marked difference in liver fat measured by histology *vs.* magnetic resonance-proton density fat fraction: A meta-analysis

Sami Qadri, Emilia Vartiainen, Mari Lahelma, Kimmo Porthan, An Tang, Ilkay S. Idilman, Jurgen H. Runge, Anne Juuti, Anne K. Penttilä, Juhani Dabek, Tiina E. Lehtimäki, Wenla Seppänen, Johanna Arola, Perttu Arkkila, Jaap Stoker, Musturay Karcaaltincaba, Michael Pavlides, Rohit Loomba, Claude B. Sirlin, Taru Tukiainen, and Hannele Yki-Järvinen

Marked difference in liver fat measured by histology *vs.* magnetic resonance-proton density fat fraction: A meta-analysis

Sami Qadri, Emilia Vartiainen, Mari Lahelma, Kimmo Porthan, An Tang, Ilkay S. Idilman, Jurgen H. Runge, Anne Juuti, Anne K. Penttilä, Juhani Dabek, Tiina E. Lehtimäki, Wenla Seppänen, Johanna Arola, Perttu Arkkila, Jaap Stoker, Musturay Karcaaltincaba, Michael Pavlides, Rohit Loomba, Claude B. Sirlin, Taru Tukiainen, Hannele Yki-Järvinen

Table of contents

Supplementary Methods 2
The Helsinki MRS-PDFF cohort 2
The liver RNA-seq cohort 3
Supplementary Tables 5
Table S1 5
Table S2 7
Table S3 8
Table S4 9
Table S5 10
Table S6 11
Table S7 12
Supplementary Figures 13
Fig. S1 13
Fig. S2 14
Fig. S3 15
Fig. S4 16
Fig. S5 17
Fig. S6 18
Fig. S7 19
Supplementary References 20

Supplementary Methods

The Helsinki MRS-PDFF cohort

Patients and study design

We recruited 71 consecutive patients undergoing metabolic surgery at the Helsinki University Hospital (Helsinki, Finland), who also underwent a liver biopsy in conjunction with liver fat measurement by MRS-PDFF. All patients fulfilled the following inclusion criteria: (i) age 18 to 75 years; (ii) no evidence of primary liver diseases other than NAFLD based on history, physical examination, and standard laboratory tests (including assays for hepatitis B virus surface antigen, hepatitis A and C virus antibodies, anti-smooth muscle antibodies, anti-mitochondrial antibodies, and anti-nuclear antibodies); (iii) alcohol consumption less than 20/30 g per day for females/males; (iv) no use of drugs or toxins associated with liver steatosis; and (v) not pregnant or lactating. Approximately a week before undergoing a liver biopsy, the patients arrived at the Clinical Research Unit after an overnight fast. A history and physical examination were performed, including measurement of body weight and height, as well as blood sampling for biochemical measurements. Spectroscopic assessment of liver fat was conducted within approximately a week of the liver biopsy. After explaining the potential risks associated with the study, all patients provided a written informed consent for their participation. The Regional Ethics Committee of the Hospital District of Helsinki and Uusimaa (Helsinki, Finland) approved the study protocol, and the study was conducted in accordance with the Declaration of Helsinki.

Liver histology

Liver biopsies were processed and stained using routine protocols of the Department of Pathology. Histopathology was assessed by an experienced hepatopathologist (J.A.), blinded to the clinical data and MRS-PDFF results.[1] Liver fat was determined as the fraction of lobular hepatocytes containing macrovesicular lipid droplets (*i.e.*, large inclusions of lipid that displace the nucleus to the cell's periphery). NASH was diagnosed when steatosis, inflammation, and ballooning were concomitantly present.[2]

Magnetic resonance spectroscopy

After a minimum of 4 hours of fasting, liver fat content was measured by ^{1}H-MRS using the 1.5T GE Signa HDxt MRI scanner (GE Medical Systems, Waukesha, WI). The point resolved spectroscopy sequence was used with an echo time (TE) of 30 ms, repetition time (TR) of 3000 ms, and with 1024 data points over 1000 kHz spectral width and 16 acquisitions. Prior

to MRS measurements, T1-weighted localization images were acquired using a standard ^1H body coil. A 27 cm^3 voxel was then carefully positioned in the right lobe of the liver avoiding large vessels, bile ducts, and the gallbladder. Subjects were allowed to breathe freely during the data collection. All spectra were analyzed by a single investigator with the jMRUI v5.2 software using the AMARES algorithm.[3] Intensities of the spectral peaks resonating from protons of water (water peak, 3–6 ppm) and protons of methylene or methyl groups in fatty acid chains (fat peak, 0.5–3 ppm) were determined using line-shape fitting with prior knowledge. Signal intensities were corrected using the equation $I_m = I_0 exp(-TE/T2)$, and T2 values of 46 ms and 58 ms were used for water and fat. Signal intensities were corrected for T2 relaxation. T1 corrections were not needed due to the long TR used. To account for the spectral complexity of liver fat, we employed an internally developed fat model which accounts for—in addition to signal originating from the fat peak—signal originating from triglyceride-associated protons underlying the water peak: the methine group peak in fatty acids (carbon–carbon double bonds with single protons, at 5.28–5.46 ppm), and the glycerol group peaks of the single proton (at 5.27 ppm) and of the double protons (at 4.22 ppm). The mathematical fat model was generated based on biochemically determined lipid composition of 125 human liver tissue samples using ultra-high performance liquid chromatography coupled with mass spectrometry. Finally, liver fat content (PDFF) was calculated as the ratio of signal from mobile protons in triglycerides to the sum of signal from mobile protons in triglycerides and free water.

The liver RNA-seq cohort

Using an identical protocol and inclusion/exclusion criteria as described above for the Helsinki MRS-PDFF cohort, we recruited a separate cohort of 138 individuals undergoing metabolic surgery at the Helsinki University Hospital. The patients similarly underwent a clinical study approximately a week of the liver biopsy. An intraoperative wedge biopsy of the liver was obtained during laparoscopy, and part of the biopsy was sent to an experienced hepatopathologist (J.A.) for histological evaluation as described above. Another part was immediately snap-frozen in liquid nitrogen for subsequent analysis of the hepatic transcriptome. The Regional Ethics Committee of the Hospital District of Helsinki and Uusimaa (Helsinki, Finland) approved the study protocol, and the study was conducted in accordance with the Declaration of Helsinki.

Liver transcriptomic analysis

Tissue RNA was extracted from the 138 liver biopsies using the AllPrep DNA/RNA/miRNA Universal Kit (QIAGEN, Hilden, Germany). Libraries were constructed using Illumina stranded mRNA library preparation (Illumina, San Diego, CA), and the samples underwent bulk RNA

sequencing using paired-end 150 bp reads on an Illumina platform. Before filtering, the number of reads was 100 M per sample. Our RNA-seq analysis pipeline closely followed the Genotype-Tissue Expression (GTEx) V8 RNA-seq analysis pipeline, with minor extensions and modifications.[4] After quality control and adapter trimming, STAR version 2.6.0a was used to align output reads to the human reference genome GRCh38/hg38.[5] Genes were annotated with STAR using GENCODE 26 transcript model annotation. Gene-level expression was calculated based on a collapsed gene model, where all isoforms were collapsed to a single transcript per gene. Read counts and transcripts per million (TPM) values were then produced using RNA-SeQC version 2.0.3.[6]

Cell-type decomposition analysis

Cell-type decomposition analysis was performed using CIBERSORTx to resolve proportions of distinct cell populations in bulk liver tissue expression profiles by using signature genes derived from a previously published human liver single-cell RNA-seq dataset.[7, 8] Default parameters were used in creating the signature matrix from single-cell RNA-seq data and computing of cell fractions using the Cell Fractions module. Batch correction was enabled with S-mode, quantile normalization was disabled, and the number of permutations was set to 100.

Supplementary Tables

Table S1. Electronic search strategy for the systematic review, which was conducted in all databases on August 16, 2022.

Search number	Query	Results
	**PubMed**	
1	"Fatty Liver"[Mesh] OR Fatty Liver*[tiab] OR Liver Fat*[tiab] OR Fat Of Liver*[tiab] OR Fat Of The Liver*[tiab] OR Fats Of Liver*[tiab] OR Fats Of The Liver*[tiab] OR Liver Steatos*[tiab] OR Steatosis Liver*[tiab] OR Steatoses Liver*[tiab] OR Steatosis Of Liver*[tiab] OR Steatosis Of The Liver*[tiab] OR Steatoses Of Liver*[tiab] OR Steatoses Of The Liver*[tiab] OR Steatohepatiti*[tiab] OR Hepatosteatos*[tiab] OR Hepatic Steatos*[tiab] OR Hepatocellular Steatos*[tiab] OR Hepatocellular Fat*[tiab] OR Hepatic Fat*[tiab] OR NAFLD[tiab] OR MAFLD[tiab] OR NASH[tiab]	73,053
2	Pathologists[Mesh] OR Pathologist*[tiab] OR Biopsy[Mesh] OR Biops*[tiab] OR Histology[Mesh] OR Histolog*[tiab]	1,441,674
3	"Magnetic Resonance Imaging"[Mesh] OR "Magnetic Resonance"[tiab] OR MRI[tiab] OR "Magnetic Resonance Spectroscopy"[Mesh] OR Spectroscop*[tiab] OR MRS[tiab] OR Magnetic Imag*[tiab] OR MR Imag*[tiab] OR PDFF[tiab] OR "Proton Density Fat Fraction"[tiab]	1,307,687
4	#1 AND #2 AND #3	1 137
	**Scopus**	
1	(TITLE-ABS-KEY("Fatty Liver*" OR "Liver* Fat*" OR "Fat* Of Liver*" OR "Fat* Of The Liver*" OR "Liver* Steatos*" OR "Steatos* Liver*" OR "Steatos* Of Liver*" OR "Steatos* Of The Liver*" OR "Steatohepatiti*" OR "Hepatosteatos*" OR "Hepatic Steatos*" OR "Hepatocellular Steatos*" OR "Hepatocellular Fat*" OR "Hepatic Fat*" OR "NAFLD" OR "MAFLD" OR "NASH")) AND (TITLE-ABS-KEY("Pathologist*" OR "Biops*" OR "Histolog*")) AND (TITLE-ABS-KEY("Magnetic Resonance" OR "MRI" OR "Spectroscop*" OR "MRS" OR "Magnetic Imag*" OR "MR Imag*" OR "PDFF" OR "Proton Density Fat Fraction"))	2 616
	**Web of Science**	
1	TS=(“Fatty Liver*” OR “Liver* Fat*” OR “Fat* Of Liver*” OR “Fat* Of The Liver*” OR “Liver* Steatos*” OR “Steatos* Liver*” OR “Steatos* Of Liver*” OR “Steatos* Of The Liver*” OR “Steatohepatiti*” OR “Hepatosteatos*” OR “Hepatic Steatos*” OR “Hepatocellular Steatos*” OR “Hepatocellular Fat*” OR “Hepatic Fat*” OR “NAFLD” OR “MAFLD” OR “NASH”)	117,285
2	TS=(“Pathologist*” OR “Biops*” OR “Histolog*”)	909,324

3	TS=(“Magnetic Resonance” OR “MRI” OR “Spectroscop*” OR “MRS” OR “Magnetic Imag*” OR “MR Imag*” OR “PDFF” OR “Proton Density Fat Fraction”)	2,610,276
4	#1 AND #2 AND #3	1 350
	**Cochrane Library**	
1	MeSH descriptor: [Fatty Liver] explode all trees	1 654
2	(“Fatty Liver*” OR “Liver* Fat*” OR “Fat* Of Liver*” OR “Fat* Of The Liver*” OR “Liver* Steatos*” OR “Steatos* Liver*” OR “Steatos* Of Liver*” OR “Steatos* Of The Liver*” OR “Steatohepatiti*” OR “Hepatosteatos*” OR “Hepatic Steatos*” OR “Hepatocellular Steatos*” OR “Hepatocellular Fat*” OR “Hepatic Fat*” OR “NAFLD” OR “MAFLD” OR “NASH”)	6 214
3	#1 OR #2	6 216
4	MeSH descriptor: [Pathologists] explode all trees	7
5	MeSH descriptor: [Biopsy] explode all trees	6 025
6	MeSH descriptor: [Histology] explode all trees	1 381
7	(“Pathologist*” OR “Biops*” OR “Histolog*”)	1 839
8	#4 OR #5 OR #6 OR #7	8 771
9	MeSH descriptor: [Magnetic Resonance Imaging] explode all trees	8 944
10	MeSH descriptor: [Magnetic Resonance Spectroscopy] explode all trees	743
11	(“Magnetic Resonance” OR “MRI” OR “Spectroscop*” OR “MRS” OR “Magnetic Imag*” OR “MR Imag*” OR “PDFF” OR “Proton Density Fat Fraction”)	46,097
12	#9 OR #10 OR #11	46,197
13	#3 AND #8 AND #12 in Trials	24

Table S2. Clinical characteristics of the Helsinki MRS-PDFF cohort.

	**All patients (n = 71)**
Age, years	52 ± 11
Males, n (%)	21 (29.6)
BMI, kg/m^2	37.6 [32.9–41.2]
fP-Glucose, mmol/L	6.4 ± 1.6
B-HbA$_{1c}$, %	6.0 ± 1.0
fP-Total cholesterol, mmol/L	4.3 ± 1.1
fP-HDL cholesterol, mmol/L	1.17 ± 0.38
fP-LDL cholesterol, mmol/L	2.8 ± 1.0
fP-Triglycerides, mmol/L	1.54 ± 1.29
P-ALT, U/L	48 ± 41
P-AST, U/L	35 ± 20
P-AST/ALT	0.9 ± 0.4
P-GGT, U/L	50 ± 58
P-ALP, U/L	73 ± 24
P-Albumin, g/L	39 ± 3
B-Platelets, E10^9/L	256 ± 57
Type 2 diabetes, n (%)	30 (42.3)
Liver fat by MRS, %	7.2 [2.8–15.7]
Liver fat by histology, %	20 [0–40]
NASH, n (%)	19 (26.7)
Fibrosis stage (F0/F1/F2/F3/F4), n	37/20/8/5/1

Data are shown as *means ± standard deviations*, *medians [25th–75th percentiles]*, *counts (percentages)*, or *counts*. Abbreviations: ALP, alkaline phosphatase; ALT, alanine aminotransferase; AST, aspartate aminotransferase; B, blood; BMI, body mass index; f, fasting; GGT, gamma-glutamyltransferase; HbA$_{1C}$, glycated hemoglobin A$_{1C}$; HDL, high-density lipoprotein; LDL, low-density lipoprotein; MRS, magnetic resonance spectroscopy; NASH, nonalcoholic steatohepatitis; P, plasma.

Table S3. Clinical characteristics of the liver RNA-seq cohort.

	**All patients (n = 138)**
Age, years	50 ± 9
Males, n (%)	46 (33.3)
BMI, kg/m^2	42.5 [37.9–46.9]
fP-Glucose, mmol/L	6.1 ± 1.3
B-HbA$_{1c}$, %	6.1 ± 1.0
fP-Total cholesterol, mmol/L	4.1 ± 1.1
fP-HDL cholesterol, mmol/L	1.15 ± 0.28
fP-LDL cholesterol, mmol/L	2.4 ± 0.9
fP-Triglycerides, mmol/L	1.51 ± 1.36
P-ALT, U/L	36 ± 23
P-AST, U/L	32 ± 14
P-AST/ALT	1.0 ± 0.4
P-GGT, U/L	39 ± 36
P-ALP, U/L	65 ± 22
P-Albumin, g/L	38 ± 3
B-Platelets, E10^9/L	251 ± 61
Type 2 diabetes, n (%)	87 (63.0)
Liver fat by histology, %	5 [0–20]
NASH, n (%)	18 (13)
Fibrosis stage (F0/F1/F2/F3/F4), n	76/49/6/6/1

Data are shown as *means ± standard deviations*, *medians [25th–75th percentiles]*, *counts (percentages)*, or *counts*. Abbreviations: ALP, alkaline phosphatase; ALT, alanine aminotransferase; AST, aspartate aminotransferase; B, blood; BMI, body mass index; f, fasting; GGT, gamma-glutamyltransferase; HbA$_{1C}$, glycated hemoglobin A$_{1C}$; HDL, high-density lipoprotein; LDL, low-density lipoprotein; NASH, nonalcoholic steatohepatitis; P, plasma.

Table S4. Methodological details of magnetic resonance protocols used in the selected studies.

Author, year, ref.	Modality	Scanner manufacturer	Field strength	Dimension	Repetition time, ms	Echo time, ms	Number of echoes	Pulse sequence	Reconstruction method	VOIs/ROIs
Qadri, 2022*	MRS-PDFF	GE	1.5 T	N/A	3000	30	1	PRESS	N/A	1 x 27 cm^3
Runge, 2018 [9]	MRS-PDFF	Philips	3.0 T	N/A	3500	10, 15, 20, 25, 30	5	STEAM	N/A	1 x 8 cm^3
Pavlides, 2017 [10]	MRS-PDFF	Siemens	3.0 T	N/A	2000	10	1	N/R	N/A	1 x 8 cm^3
Traussnigg, 2017 [11]	MRS-PDFF	Siemens	3.0 T	N/A	3000	30	1	PRESS	N/A	1 x 27 cm^3
Rastogi, 2016 [12]	MRS-PDFF	Philips	3.0 T	N/A	2000	15, 20, 25, 30, 35	5	STEAM	N/A	1 x 8 cm^3
Tang, 2015 [13]	MRI-PDFF	GE	3.0 T	2D	2000	1.15–6.9	6	N/A	Magnitude	9 x ~3.14 cm^2
Hwang, 2014 [14]	MRS-PDFF	Siemens	3.0 T	N/A	3000	12, 24, 36, 48, 72	5	STEAM	N/A	1 x 27 cm^3
Parente, 2014 [15]	MRS-PDFF	Philips	3.0 T	N/A	2000 / 4000	40, 50, 60, 70, 80, 90, 100, 110	8	PRESS	N/A	1 x 27 cm^3
Idilman, 2013 [16]	MRI-PDFF	GE	1.5 T	3D	3000	1.6–9.8	6	N/A	Complex	1 x 4 cm^2 + 8 x 2 cm^2

Abbreviations: N/A, not applicable; N/R, not reported; PRESS, point-resolved spectroscopy; STEAM, stimulated echo acquisition mode.
*Previously unpublished data from the Helsinki MRS-PDFF cohort.

Table S5. QUADAS-2 quality and risk-of-bias assessment.

Study	Risk of bias				Applicability concerns		
	Patient selection	Index test	Reference standard	Flow and timing	Patient selection	Index test	Reference standard
Qadri 2022	☺	☺	☹	☺	☺	☺	☺
Runge 2018	☺	☺	☹	☺	☺	☺	☺
Pavlides 2017	?	☺	☺	☺	☺	☺	☺
Traussnigg 2017	☺	☺	?	☺	☺	☺	☺
Rastogi 2016	?	☺	☹	☺	☺	☺	☺
Tang 2015	☺	☺	☹	☺	☺	☺	☺
Parente 2014	☺	☺	☹	☺	☺	☺	☺
Hwang 2014	☺	☺	☹	☺	☺	☺	☺
Idilman 2013	☺	☺	☹	☺	☺	☺	☺

☺ Low Risk ☹ High Risk ? Unclear Risk

Table S6. Classification of steatosis grades by histology as compared with PDFF, based on thresholds that are commonly used for histological steatosis grade assessment.

	PDFF				
**Histology**	**S0**	**S1**	**S2**	**S3**	**Total**
**S0**	119	19	0	0	138
**S1**	66	182	0	0	248
**S2**	2	112	2	0	116
**S3**	0	84	11	0	95
**Total**	187	397	13	0	597

Thresholds used to classify steatosis grades: S0: <5%; S1: 5–33%; S2: 34–66%; S3: >66%.

Table S7. Thresholds and raw diagnostic performance parameters for PDFF to predict dichotomized histological steatosis grades at varying sensitivities and specificities in the pooled cohort.

Steatosis grade classification	Threshold	Se, % (95% CI)	Sp, % (95% CI)	PPV, % (95% CI)	NPV, % (95% CI)
**Specificity 90% (rule-in thresholds)**					
S0 vs. S1–S3	≥5.75	79.5 (75.8–83.0)	90.6 (85.5–94.9)	96.6 (94.8–98.2)	57.1 (52.9–61.8)
S0–S1 vs. S2–S3	≥15.50	78.7 (73.0–84.4)	90.2 (87.1–93.0)	81.4 (76.8–86.1)	88.5 (85.9–91.3)
S0–S2 vs. S3	≥21.35	69.5 (60.0–77.9)	90.0 (87.5–92.6)	56.9 (49.6–64.6)	94.0 (92.2–95.7)
**Specificity 95% (rule-in thresholds)**					
S0 vs. S1–S3	≥6.49	76.9 (72.8–80.8)	95.6 (92.0–98.5)	98.3 (97.0–99.4)	55.5 (51.4–60.1)
S0–S1 vs. S2–S3	≥18.52	63.5 (57.3–69.7)	95.1 (92.7–97.1)	87.7 (82.5–92.5)	82.7 (80.3–85.2)
S0–S2 vs. S3	≥25.15	50.5 (41.0–61.0)	95.0 (93.0–96.8)	65.8 (56.2–75.8)	91.1 (89.4–92.8)
**Sensitivity 90% (rule-out thresholds)**					
S0 vs. S1–S3	<4.11	90.2 (87.6–92.8)	81.2 (74.6–87.7)	94.1 (92.2–96.1)	71.4 (65.6–77.5)
S0–S1 vs. S2–S3	<11.49	90.0 (85.8–93.8)	81.3 (77.5–85.5)	72.5 (68.6–77.1)	93.7 (91.3–96.1)
S0–S2 vs. S3	<15.06	90.5 (84.2–95.8)	73.7 (69.9–77.5)	39.5 (36.0–43.4)	97.6 (96.1–99.0)
**Sensitivity 95% (rule-out thresholds)**					
S0 vs. S1–S3	<2.53	95.2 (93.0–96.9)	53.6 (45.6–61.6)	87.2 (85.3–89.2)	77.4 (69.2–84.8)
S0–S1 vs. S2–S3	<10.17	95.3 (92.4–97.6)	75.6 (71.2–80.0)	68.1 (64.3–72.4)	96.7 (94.6–98.4)
S0–S2 vs. S3	<11.39	95.8 (91.6–98.9)	65.7 (61.3–70.1)	34.6 (31.7–37.9)	98.8 (97.6–99.7)

Abbreviations: Se, sensitivity; Sp, specificity; PPV, positive predictive value; NPV, negative predictive value; CI, confidence interval.

Supplementary Figures

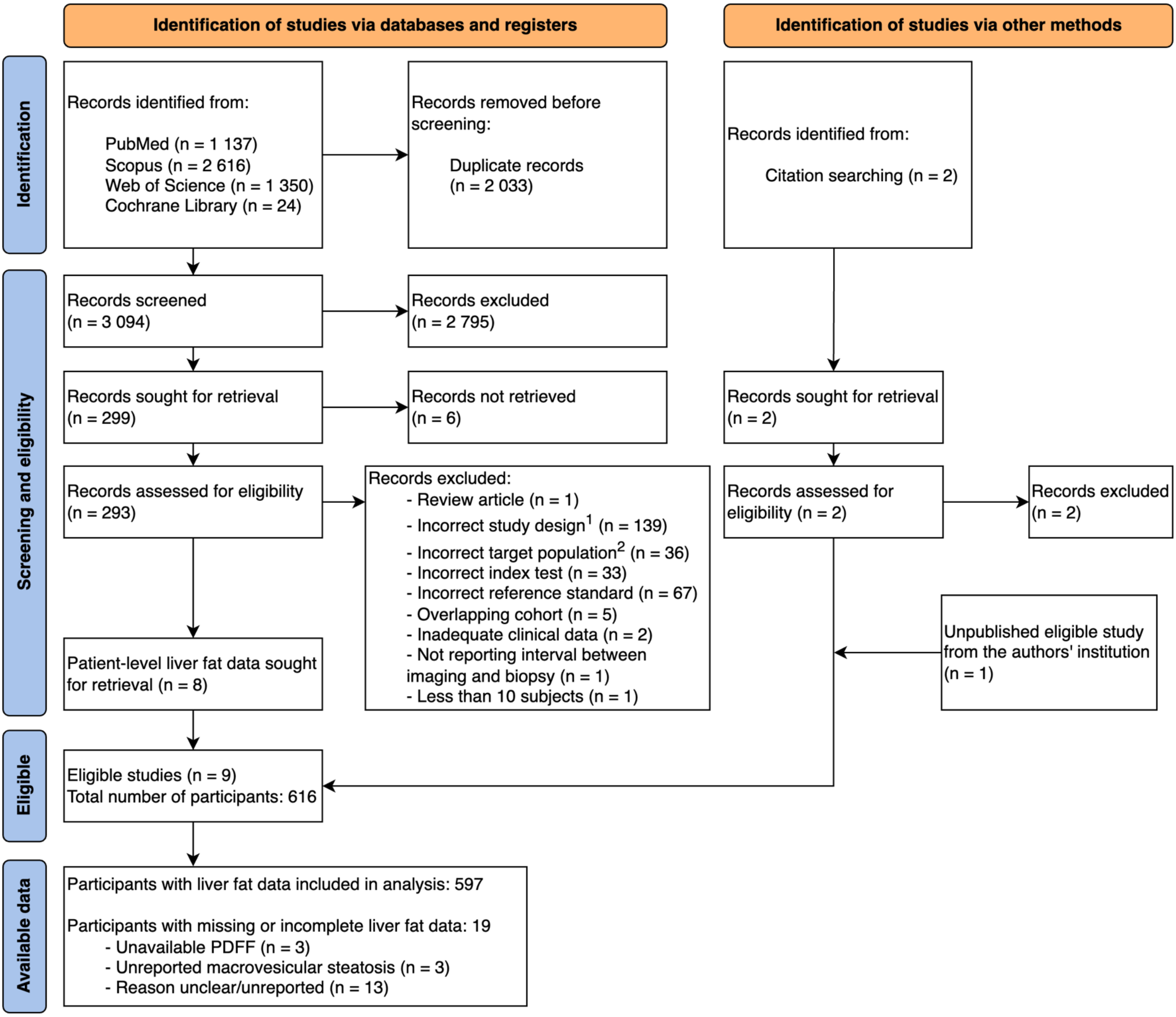

[1]Including studies not reporting data on associations between histological steatosis and PDFF.
[2]Including studies conducted in pediatric populations, with animals, or *ex vivo*, as well as studies including patients with primary liver diseases other than NAFLD, with liver cancer, or with metastases.

Adopted from: Page MJ, McKenzie JE, Bossuyt PM, Boutron I, Hoffmann TC, Mulrow CD, et al. The PRISMA 2020 statement: an updated guideline for reporting systematic reviews. BMJ 2021;372:n71. doi: 10.1136/bmj.n71. For more information, visit: http://www.prisma-statement.org/

Fig. S1. PRISMA flow diagram for the study selection.

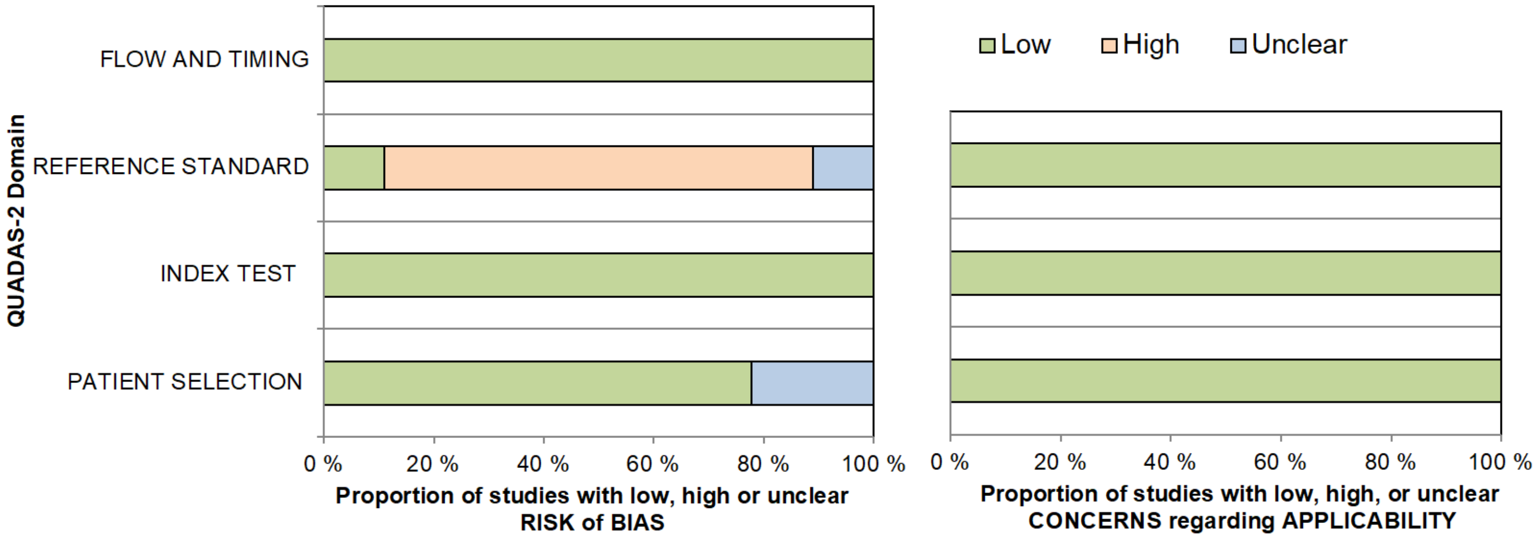

Fig. S2. QUADAS-2 quality and risk-of-bias assessment.

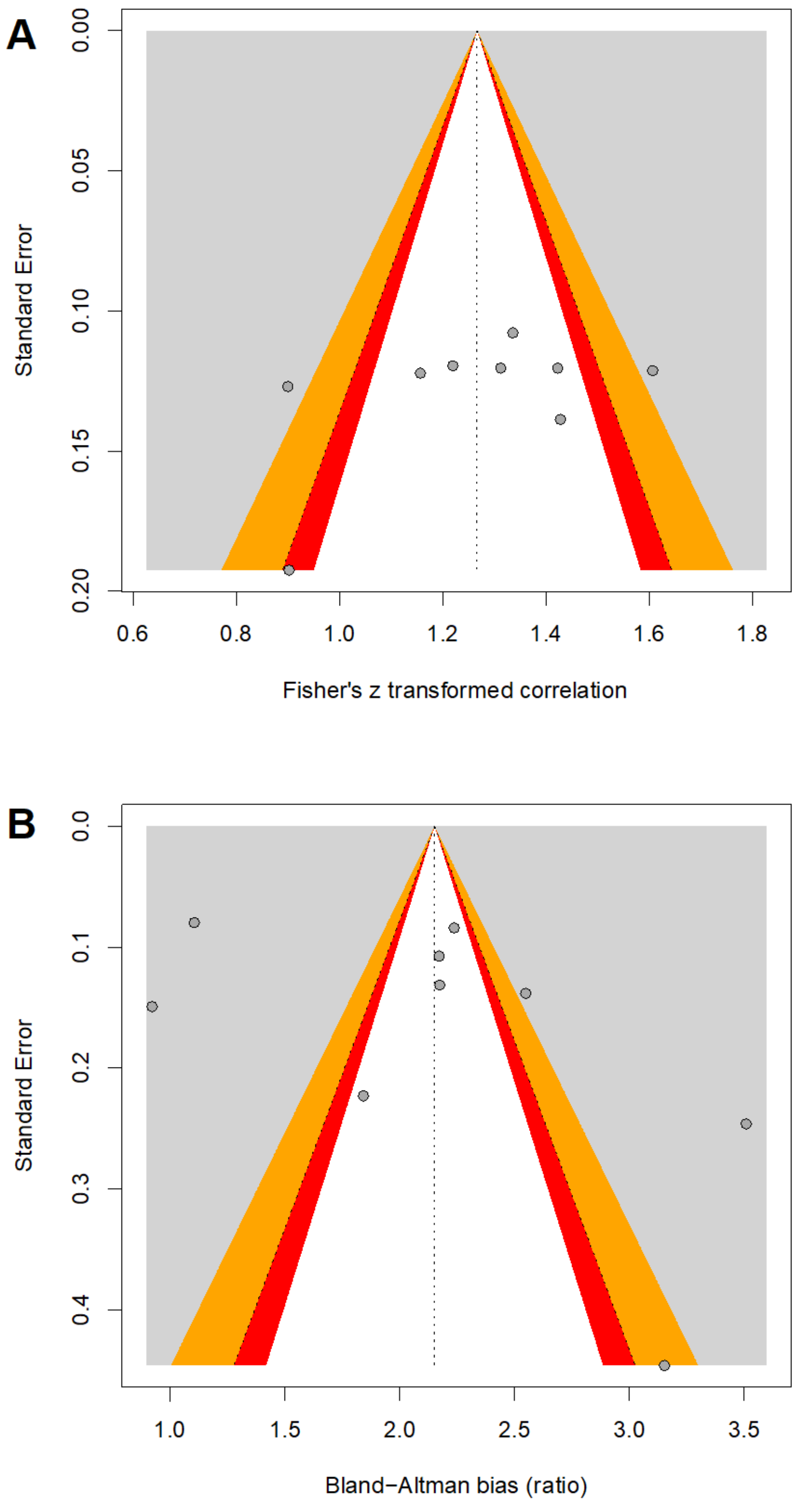

Fig. S3. Contour-enhanced funnel plots showing distribution of the included studies with respect to their (A) Fisher's z transformed Pearson correlation coefficients and their standard errors and (B) proportional Bland-Altman bias estimates and their standard errors. Red zones denote 90–95% confidence limits, while orange zones denote 95–99% confidence limits.

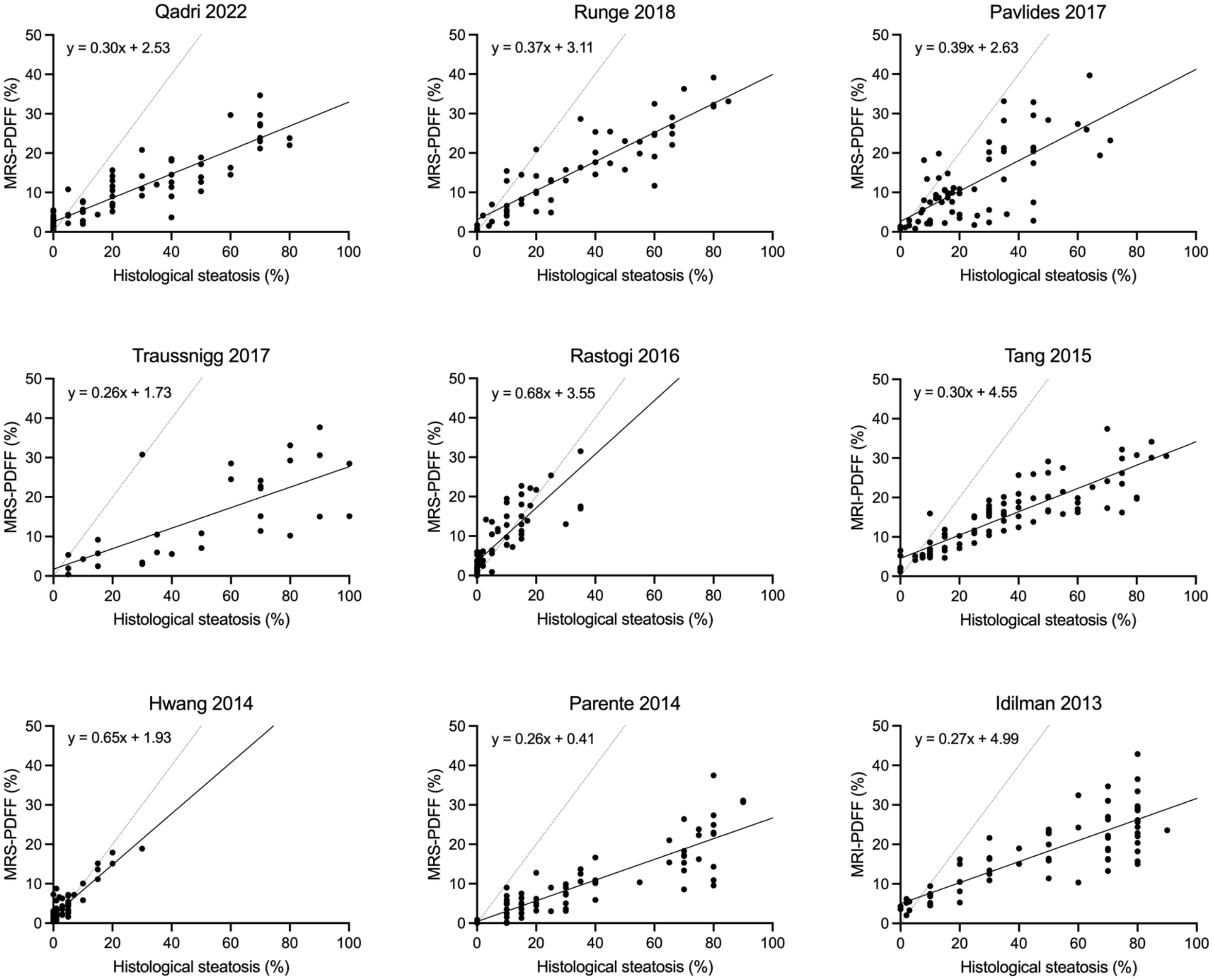

Fig. S4. Relationship between histological steatosis and PDFF in the individual studies. Lines were fit using linear regression. Dotted gray line is the line of identity.

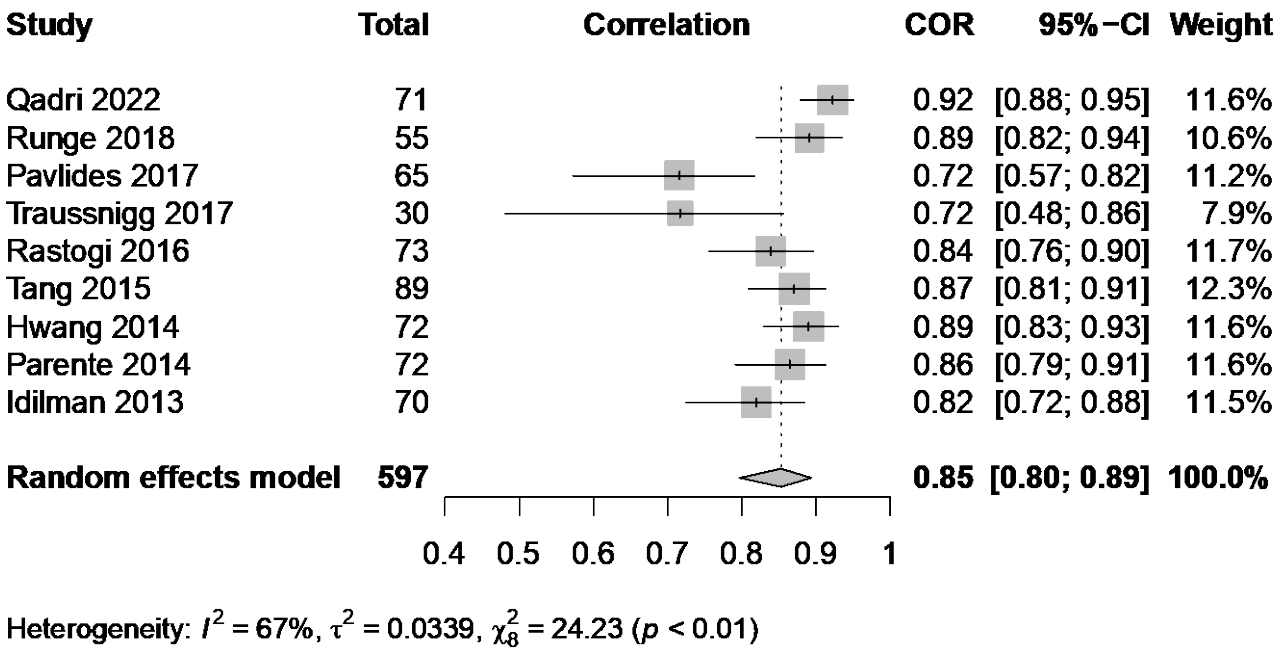

Fig. S5. Random-effects meta-analysis of Pearson correlation coefficients for histological steatosis and PDFF in the included studies.

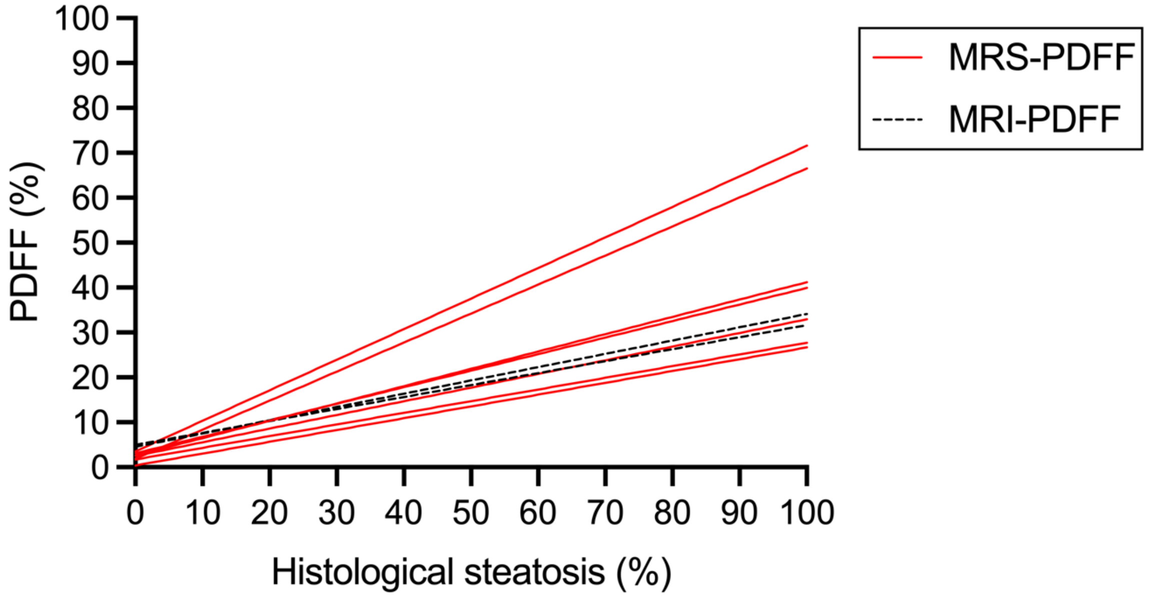

Fig. S6. Linear regression lines for the individual studies showing associations between histological steatosis and PDFF. Solid red lines denote studies using MRS-PDFF, and dashed black lines denote studies using MRI-PDFF.

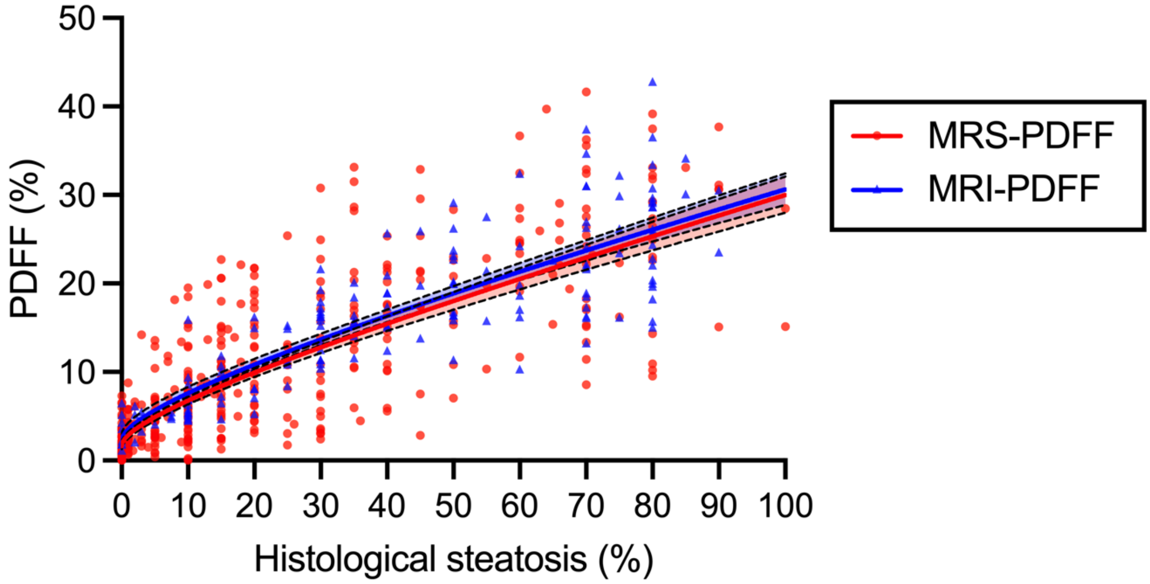

Fig. S7. Relationship between histological steatosis and PDFF, stratified by use of either MRS-PDFF or MRI-PDFF. The solid red circles and the red line denote MRS-PDFF, and the solid blue triangles and the blue line denote MRI-PDFF. The best-fit lines were determined using linear regression. Both variables underwent square root transformation prior to model fitting, and the curve fit was then backtransformed for display. The shaded areas denote 95% CI.

Supplementary References

[1] Bedossa P, Poitou C, Veyrie N, Bouillot JL, Basdevant A, Paradis V, et al. Histopathological algorithm and scoring system for evaluation of liver lesions in morbidly obese patients. Hepatology 2012;56:1751-1759.

[2] Bedossa P, FLIP Pathology Consortium. Utility and appropriateness of the fatty liver inhibition of progression (FLIP) algorithm and steatosis, activity, and fibrosis (SAF) score in the evaluation of biopsies of nonalcoholic fatty liver disease. Hepatology 2014;60:565-575.

[3] Vanhamme L, van den Boogaart A, Van Huffel S. Improved method for accurate and efficient quantification of MRS data with use of prior knowledge. Journal of magnetic resonance 1997;129:35-43.

[4] The Genotype-Tissue Expression (GTEx) Consortium. GTEx V8 Analysis Methods. Updated 2019 Aug 20 [cited 2022 Sept 12]; Available from: https://gtexportal.org/home/methods

[5] Dobin A, Davis CA, Schlesinger F, Drenkow J, Zaleski C, Jha S, et al. STAR: ultrafast universal RNA-seq aligner. Bioinformatics 2013;29:15-21.

[6] DeLuca DS, Levin JZ, Sivachenko A, Fennell T, Nazaire MD, Williams C, et al. RNA-SeQC: RNA-seq metrics for quality control and process optimization. Bioinformatics 2012;28:1530-1532.

[7] Newman AM, Steen CB, Liu CL, Gentles AJ, Chaudhuri AA, Scherer F, et al. Determining cell type abundance and expression from bulk tissues with digital cytometry. Nat Biotechnol 2019;37:773-782.

[8] MacParland SA, Liu JC, Ma XZ, Innes BT, Bartczak AM, Gage BK, et al. Single cell RNA sequencing of human liver reveals distinct intrahepatic macrophage populations. Nat Commun 2018;9:4383.

[9] Runge JH, Smits LP, Verheij J, Depla A, Kuiken SD, Baak BC, et al. MR Spectroscopy-derived Proton Density Fat Fraction Is Superior to Controlled Attenuation Parameter for Detecting and Grading Hepatic Steatosis. Radiology 2018;286:547-556.

[10] Pavlides M, Banerjee R, Tunnicliffe EM, Kelly C, Collier J, Wang LM, et al. Multiparametric magnetic resonance imaging for the assessment of non-alcoholic fatty liver disease severity. Liver Int 2017;37:1065-1073.

[11] Traussnigg S, Kienbacher C, Gajdosik M, Valkovic L, Halilbasic E, Stift J, et al. Ultra-high-field magnetic resonance spectroscopy in non-alcoholic fatty liver disease: Novel mechanistic and diagnostic insights of energy metabolism in non-alcoholic steatohepatitis and advanced fibrosis. Liver Int 2017;37:1544-1553.

[12] Rastogi R, Gupta S, Garg B, Vohra S, Wadhawan M, Rastogi H. Comparative accuracy of CT, dual-echo MRI and MR spectroscopy for preoperative liver fat quantification in living related liver donors. Indian J Radiol Imaging 2016;26:5-14.

[13] Tang A, Desai A, Hamilton G, Wolfson T, Gamst A, Lam J, et al. Accuracy of MR imaging-estimated proton density fat fraction for classification of dichotomized histologic steatosis grades in nonalcoholic fatty liver disease. Radiology 2015;274:416-425.

[14] Hwang I, Lee JM, Lee KB, Yoon JH, Kiefer B, Han JK, Choi BI. Hepatic steatosis in living liver donor candidates: preoperative assessment by using breath-hold triple-echo MR imaging and 1H MR spectroscopy. Radiology 2014;271:730-738.

[15] Parente DB, Rodrigues RS, Paiva FF, Oliveira Neto JA, Machado-Silva L, Lanzoni V, et al. Is MR spectroscopy really the best MR-based method for the evaluation of fatty liver in diabetic patients in clinical practice? PLoS One 2014;9:e112574.

[16] Idilman IS, Aniktar H, Idilman R, Kabacam G, Savas B, Elhan A, et al. Hepatic steatosis: quantification by proton density fat fraction with MR imaging versus liver biopsy. Radiology 2013;267:767-775.
